# USP47 inhibits m^6^A-dependent c-Myc translation to maintain regulatory T cell metabolic and functional homeostasis

**DOI:** 10.1172/JCI169365

**Published:** 2023-12-01

**Authors:** Aiting Wang, Haiyan Huang, Jian-Hong Shi, Xiaoyan Yu, Rui Ding, Yuerong Zhang, Qiaoqiao Han, Zhi-Yu Ni, Xia Li, Ren Zhao, Qiang Zou

**Affiliations:** 1 Department of General Surgery, Ruijin Hospital, and; 2 Shanghai Institute of Immunology, Department of Immunology and Microbiology, State Key Laboratory of Systems Medicine for Cancer, Shanghai Jiao Tong University School of Medicine, Shanghai, China.; 3 Center for Cancer Immunology, Institute of Biomedicine and Biotechnology, Shenzhen Institute of Advanced Technology, Chinese Academy of Sciences, Shenzhen, Guangdong Province, China.; 4 Central Laboratory, Hebei Collaborative Innovation Center of Tumor Microecological Metabolism Regulation, Affiliated Hospital of Hebei University, Baoding, Hebei Province, China.; 5 Innovative Institute of Chinese Medicine and Pharmacy, Shandong University of Traditional Chinese Medicine, Jinan, Shandong Province, China.

**Keywords:** Autoimmunity, Immunology, Adaptive immunity

## Abstract

The functional integrity of Tregs is interwoven with cellular metabolism; however, the mechanisms governing Treg metabolic programs remain elusive. Here, we identified that the deubiquitinase USP47 inhibited c-Myc translation mediated by the RNA *N*^6^-methyladenosine (m^6^A) reader YTHDF1 to maintain Treg metabolic and functional homeostasis. USP47 positively correlated with the tumor-infiltrating Treg signature in samples from patients with colorectal cancer and gastric cancer. USP47 ablation compromised Treg homeostasis and function in vivo, resulting in the development of inflammatory disorders, and boosted antitumor immune responses. USP47 deficiency in Tregs triggered the accumulation of the c-Myc protein and in turn exacerbated hyperglycolysis. Mechanistically, USP47 prevented YTHDF1 ubiquitination to attenuate the association of YTHDF1 with translation initiation machinery, thereby decreasing m^6^A-based c-Myc translation efficiency. Our findings reveal that USP47 directs m^6^A-dependent metabolic programs to orchestrate Treg homeostasis and suggest novel approaches for selective immune modulation in cancer and autoimmune diseases by targeting of USP47.

## Graphical abstract

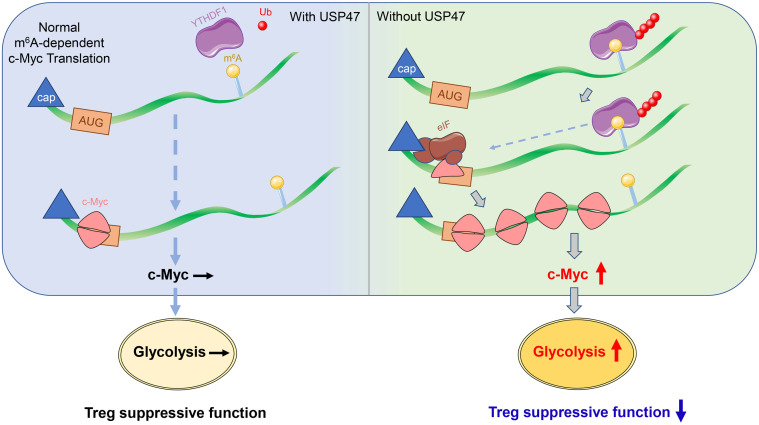

## Introduction

Tregs are essential for immune tolerance and play a critical role in preventing autoimmune inflammation and associated pathologies (1–3). Tregs also drive immunosuppression to dampen antitumor immune responses in the tumor microenvironment (4, 5). The function and fate determination of Tregs is tightly regulated by cellular metabolic pathways (6–8). Mitochondrial oxidative metabolism, not glycolysis, is a main driver of Treg function and homeostasis (9, 10). Excessive and sustained glycolysis leads to Treg instability, although glycolysis activation is required for Treg migration (11–13). However, how the Treg metabolic networks are rewired to support their functional adaptation remains unclear.

c-Myc acts as a master transcriptional regulator and is involved in Treg metabolism and homeostasis (14–16). c-Myc is indispensable for Treg function because it regulates mitochondrial oxidative metabolism (15). Therefore, loss of c-Myc in Tregs results in early-onset autoimmune disorders and uncontrolled T cell and germinal center responses (15). On the other hand, elevated c-Myc expression induces excessive glycolysis, which compromises Treg survival and function (11). Autophagy inhibits mTORC1 activation to inhibit c-Myc expression and glycolytic metabolism and maintain the survival and stability of Tregs (11, 17). Pharmacological inhibition of mTORC1, c-Myc, or glycolytic activity restores the stability of autophagy-deficient Tregs (11). These findings suggest that a moderate level of c-Myc protein expression benefits Treg metabolic programs and functional integrity. Notably, the molecular mechanism governing c-Myc expression in Tregs has not been reported.

*N*^6^-methyladenosine (m^6^A) is one of the most prevalent post-transcriptional modifications of eukaryotic mRNA and exerts important effects on mRNA splicing, stability, and translation (18). A methyl group is dynamically deposited on mRNA at *N*^6^ adenosine position, by m^6^A methyltransferases, and subsequently erased by demethylases and is recognized by m^6^A reader proteins (19). m^6^A-dependent gene regulation is crucial for innate and adaptive cell-mediated immunity (20–24). Deletion of the m^6^A reader protein YTHDF1 in dendritic cells (DCs) enhanced DC cross-priming ability and induced potent antitumor immune responses (25). Loss of the m^6^A writer protein METTL3 in T cells disrupted T cell homeostatic proliferation and differentiation (26). In addition, METTL3 sustained Treg suppression of autoimmunity (27). However, it is still unknown whether and how the m^6^A modification regulates metabolic programs in different types of immune cells, especially in Tregs.

Deubiquitinases (DUBs) have been identified as being involved in Treg biology. USP7- or USP21-mediated deubiquitination promotes Foxp3 stabilization to increase Treg suppressive capacity (28, 29). CRISPR screen in Tregs reveals that USP22 stabilizes Foxp3 expression to maintain Treg suppressive function (30). USP44 cooperates with USP7 to mediate the deubiquitination and stabilization of Foxp3, thus modulating Treg function (31). USP1 promotes the proteasomal degradation of Foxp3 by deubiquitinating and stabilizing TAZ, thereby attenuating Treg differentiation (32). In this study, we show that USP47 directs YTHDF1-dependent c-Myc translation to maintain Treg metabolic and functional homeostasis. USP47 was positively correlated with the tumor-infiltrating Treg signature in samples from patients with colorectal cancer (CRC) and gastric cancer (GC). Treg-specific deletion of USP47 resulted in autoimmune disorders, severe colonic inflammation, and enhanced antitumor immunity in mice. USP47-deficient Tregs exhibited a transcriptional defect that compromised in vivo homeostasis and function. Loss of USP47 led to excessive c-Myc activity and hyperglycolysis, which was responsible for defective Treg homeostasis and function. Mechanistically, Treg-derived USP47 mediated YTHDF1 deubiquitination to attenuate the association of YTHDF1 with translation initiation machinery and inhibit m^6^A-based c-Myc translation. Our results highlight the importance of m^6^A-dependent metabolic reprogramming for orchestrating Treg homeostasis and function in the establishment of immune tolerance.

## Results

### USP47 positively correlates with the intratumoral Treg signature.

**Figure 1:**
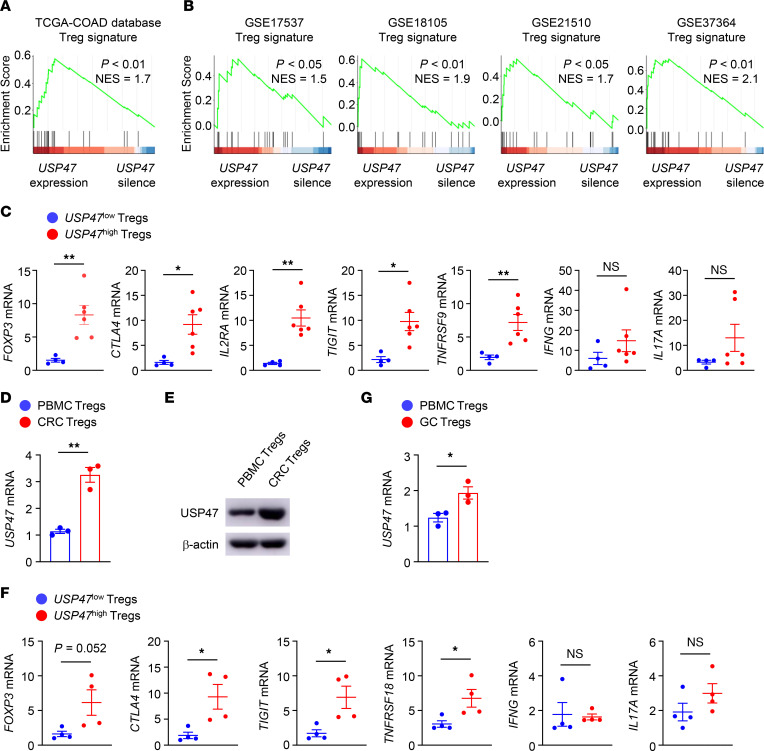
USP47 positively correlates with intratumoral Treg signature. (**A**) Gene set enrichment analysis (GSEA) of Treg signature genes in USP47-expressing and USP47-silenced Tregs from The Cancer Genome Atlas (TCGA)–colon adenocarcinoma (COAD) database. NES, normalization enrichment score. (**B**) GSEA of Treg signature genes in USP47-expressing and USP47-silenced Tregs from 4 GEO data sets for COAD. (**C**) qRT-PCR analysis of Treg signature genes in tumor-infiltrating Tregs from CRC. The normalized *USP47* expression value of tumor-infiltrating Tregs with the lowest expression of *USP47* was set to be 1. The normalized *USP47* expression values of *USP47^hi^* Tregs were more than 3 (*n* = 6), and those of *USP47^lo^* Tregs were less than 3 (*n* = 4). (**D** and **E**) qRT-PCR analysis of *USP47* mRNA levels (**D**; *n* = 3) and immunoblot analysis of USP47 expression (**E**) in Tregs from PBMCs and CRC tissues. (**F**) qRT-PCR analysis of Treg signature genes in tumor-infiltrating Tregs from GC. The normalized *USP47* expression value of tumor-infiltrating Tregs with the lowest expression of *USP47* was set to be 1. The normalized *USP47* expression values of *USP47^hi^* Tregs were more than 3 (*n* = 4), and those of *USP47^lo^* Tregs were less than 3 (*n* = 4). (**G**) qRT-PCR analysis of *USP47* mRNA levels in Tregs from PBMCs and GC tissues (*n* = 3). Representative data are shown from 3 independent experiments (**C**–**G**). Data are represented as mean ± SEM. *P < 0.05; **P < 0.01. Two-tailed Student’s *t* test (**C**, **D**, **F**, and **G**).

To identify the critical regulator modulating Treg identity in the tumor microenvironment, we analyzed the gene expression profiles of tumor-infiltrating Tregs in The Cancer Genome Atlas colon adenocarcinoma (COAD) data set (33). Interestingly, we observed a significantly positive correlation between the Treg signature and USP47 expression in COAD patients (Figure 1A). Using the Gene Expression Omnibus (GEO) data sets for COAD (34–37), we found that USP47-expressing Tregs from CRC tissues exhibited enrichment with Treg signature genes (Figure 1B), indicating that the Treg signature was associated with higher expression levels of USP47 in the tumor-infiltrating Tregs. To validate these findings, we used Tregs from human CRC tissue samples for quantitative reverse transcription PCR (qRT-PCR) analysis. Indeed, the expression of Treg signature genes was upregulated in *USP47^hi^* Tregs from CRC tissue (Figure 1C). Moreover, the mRNA and protein levels of USP47 in Tregs from human CRC tissues were higher than those in Tregs in a population of peripheral blood mononuclear cells (PBMCs) (Figure 1, D and E). Parallel studies using Tregs from GC patients confirmed that *USP47^hi^* Tregs showed increased expression levels of Treg signature genes (Figure 1F). Furthermore, Tregs from GC tissues showed significantly higher mRNA levels of *USP47* than those in the PBMC population (Figure 1G). In contrast, non-Treg CD4^+^ or CD8^+^ T cells from CRC or GC tissues and those in the PBMC population displayed similar mRNA and protein levels of USP47 (Supplemental Figure 1, A–F; supplemental material available online with this article; https://doi.org/10.1172/JCI169365DS1). These results suggest that USP47 positively correlates with the Treg signature in tumor-infiltrating Tregs.

### USP47 is required for the maintenance of Treg transcriptional programs.

**Figure 2:**
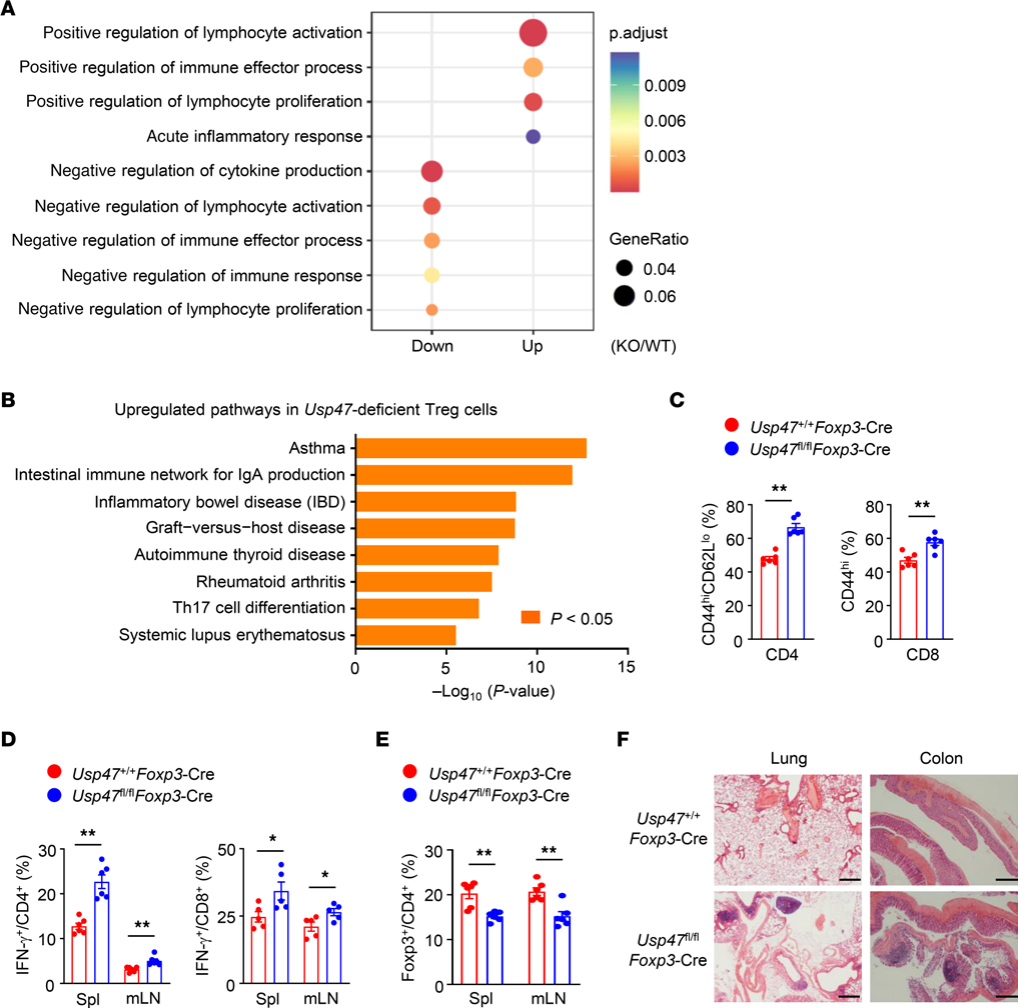
USP47 maintains Treg transcriptional programs. (**A**) Gene Ontology enrichment analysis of downregulated (Down) and upregulated (Up) gene sets in *Usp47*-deficient Tregs (KO) compared with wild-type Tregs (WT) stimulated with anti-CD3 and anti-CD28 for 24 hours. (**B**) Functional enrichment analysis of Kyoto Encyclopedia of Genes and Genomes (KEGG) pathways upregulated in *Usp47*-deficient Tregs compared with WT Tregs. (**C**) Flow cytometric analysis of the proportions of effector/memory-like CD44^hi^CD62L^lo^CD4^+^ T cells and CD44^hi^CD8^+^ T cells in the spleen from 8- to 10-month-old *Usp47^+/+^Foxp3*-Cre and *Usp47^fl/fl^Foxp3*-Cre mice (*n* = 6). (**D**) Flow cytometric analysis of the percentages of IFN-γ–producing CD4^+^ T cells (*n* = 6) and CD8^+^ T cells (*n* = 5) in the spleen (Spl) and mesenteric lymph nodes (mLN) from 8- to 10-month-old *Usp47^+/+^Foxp3*-Cre and *Usp47^fl/fl^Foxp3*-Cre mice. (**E**) Flow cytometric analysis of the proportions of CD4^+^Foxp3^+^ Tregs in the spleen and mLNs from 8- to 10-month-old *Usp47^+/+^Foxp3*-Cre and *Usp47^fl/fl^Foxp3*-Cre mice (*n* = 6). (**F**) H&E staining of the indicated tissue sections of 10-month-old *Usp47^+/+^Foxp3*-Cre and *Usp47^fl/fl^Foxp3*-Cre mice. Scale bars: 500 μm. Data are representative of 3 independent experiments (**C**–**F**). Data are presented as mean ± SEM. *P < 0.05; **P < 0.01. Two-tailed Student’s *t* test (**C**–**E**).

To explore the role of USP47 in Tregs, we crossed *Usp47^fl/fl^* mice with *Foxp3-*YFP-Cre mice to obtain *Usp47* Treg–conditional knockout (*Usp47^fl/fl^Foxp3*-Cre) mice (Supplemental Figure 2A). Six-week-old *Usp47^fl/fl^Foxp3*-Cre mice did not exhibit obvious abnormalities in thymocyte development, peripheral T cell homeostasis, or Treg frequency (Supplemental Figure 2, B–D). However, the T cell homeostasis was affected in 10-week-old and 3-month-old *Usp47^fl/fl^Foxp3*-Cre mice (Supplemental Figure 2, E and F). We then assessed the potential importance of USP47 in Treg transcriptional programs by performing a transcriptomic analysis of *Usp47^+/+^Foxp3*-Cre and *Usp47^fl/fl^Foxp3*-Cre mouse Tregs stimulated with anti-CD3 and anti-CD28 antibodies plus IL-2 for 24 hours. A Gene Ontology analysis revealed that negative regulation of T cell activation, proliferation, or effector cytokine production was downregulated in the USP47-deficient Tregs (Figure 2A). In contrast, positive regulation of effector T cell functions was upregulated in USP47-deficient Tregs (Figure 2A). Furthermore, the significant upregulation of autoimmune disease–associated gene expression in USP47-deficient Tregs was observed in a functional pathway enrichment analysis (Figure 2B), indicating a transcriptional defect in USP47-deficient Tregs. Consistent with these alterations in transcriptional networks, 10-month-old *Usp47^fl/fl^Foxp3*-Cre mice displayed considerable disruption to T cell homeostasis (Figure 2, C and D), with a substantially reduced Treg population (Figure 2E). Histological examination revealed an extensive lymphoid inflammatory presence in peripheral organs (Figure 2F). These results suggest that USP47 is required for the maintenance of Treg transcriptional programs to control immune homeostasis.

### USP47 ablation dampens Treg immunosuppressive functions in vivo.

**Figure 3:**
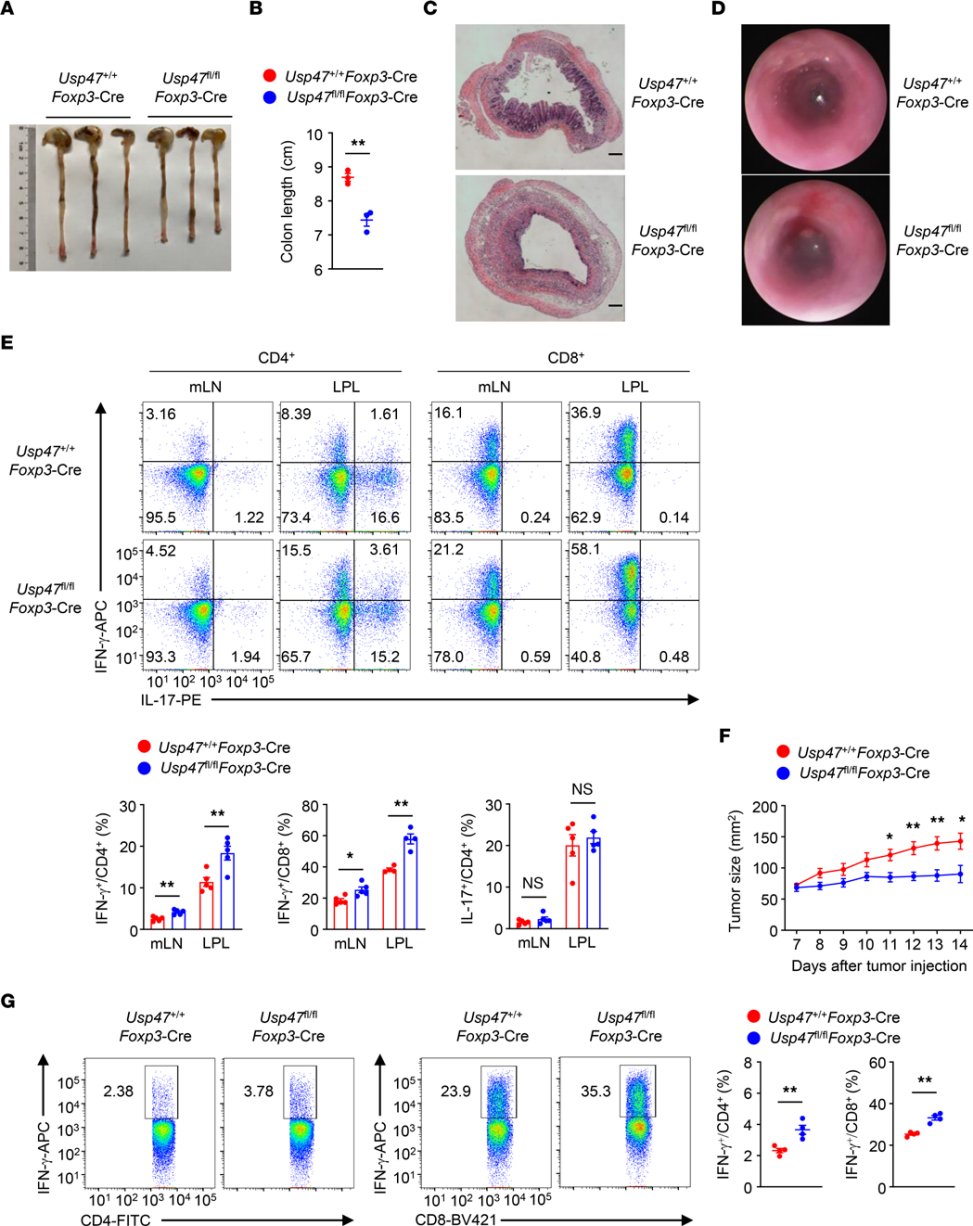
USP47 ablation dampens Treg functions in vivo. (**A**–**E**) Three-month-old *Usp47^+/+^Foxp3*-Cre and *Usp47^fl/fl^Foxp3*-Cre mice were provided with drinking water containing 2.5% DSS for 7 days, followed by regular water. Mice were killed at day 9. They were analyzed for colon length (**A** and **B**; *n* = 3), H&E staining of colon section (**C**; scale bars: 500 μm), and images of mini-endoscopic colon (**D**). Flow cytometric analysis of the proportions of IFN-γ– or IL-17–producing CD4^+^ and CD8^+^ T cells in the mLNs and lamina propria lymphocytes (LPL) from mice after DSS-induced colitis (**E**; *n* = 4 or 5). (**F** and **G**) Ten-week-old *Usp47^+/+^Foxp3*-Cre and *Usp47^fl/fl^Foxp3*-Cre mice were injected subcutaneously with MC38 colon cancer cells, and 15 days after injection, mice were sacrificed. Tumor growth in 10-week-old *Usp47^+/+^Foxp3*-Cre and *Usp47^fl/fl^Foxp3*-Cre mice injected with MC38 colon cancer cells (**F**; *n* = 5). Flow cytometric analysis of the percentages of IFN-γ–producing CD4^+^ and CD8^+^ T cells in the tumors from *Usp47^+/+^Foxp3*-Cre and *Usp47^fl/fl^Foxp3*-Cre mice injected with MC38 tumor cells (**G**; *n* = 4). Representative data are shown from 3 independent experiments. Data are presented as mean ± SEM. *P < 0.05; **P < 0.01. Two-tailed Student’s *t* test (**B**, **E**–**G**).

Because our transcriptomic analysis revealed upregulated pathways of chronic inflammatory diseases including inflammatory bowel disease (IBD) in USP47-deficient Tregs (Figure 2B), we assessed the colonic inflammatory responses of *Usp47^+/+^Foxp3*-Cre and *Usp47^fl/fl^Foxp3*-Cre mice using a dextran sodium sulfate–induced (DSS-induced) acute colitis model. The DSS-treated *Usp47^fl/fl^Foxp3*-Cre mice displayed exacerbated colon length shortening (Figure 3, A and B) and hyperplasia of the colonic mucosa (Figure 3C). A mini-endoscopic analysis showed that the *Usp47^fl/fl^Foxp3*-Cre mice exhibited more colonic bleeding than the wild-type (WT) mice (Figure 3D). Furthermore, a greater frequency of memory and effector-like T cells was observed in the DSS-treated *Usp47^fl/fl^Foxp3*-Cre mice (Figure 3E), suggesting considerable attenuation of the suppressive activity of USP47-deficient Tregs. Given that USP47 correlated with the Treg signature in human CRC and GC tissues, we inoculated *Usp47^+/+^Foxp3*-Cre and *Usp47^fl/fl^Foxp3*-Cre mice with MC38 murine colon cancer cells to examine the functional importance of USP47 in tumor-infiltrating Tregs. Compared with the WT mice, the *Usp47^fl/fl^Foxp3*-Cre mice exhibited a profound decrease in tumor size (Figure 3F). In addition, T cells in the tumors of the tumor-bearing *Usp47^fl/fl^Foxp3*-Cre mice produced higher levels of IFN-γ than those from the tumor-bearing WT mice (Figure 3G). Therefore, Treg-specific USP47 deletion exacerbated experimental colitis and boosted antitumor immune responses, indicating that USP47 is indispensable for Treg immunosuppressive functions in vivo.

### USP47 maintains Treg homeostasis and function in vivo.

**Figure 4:**
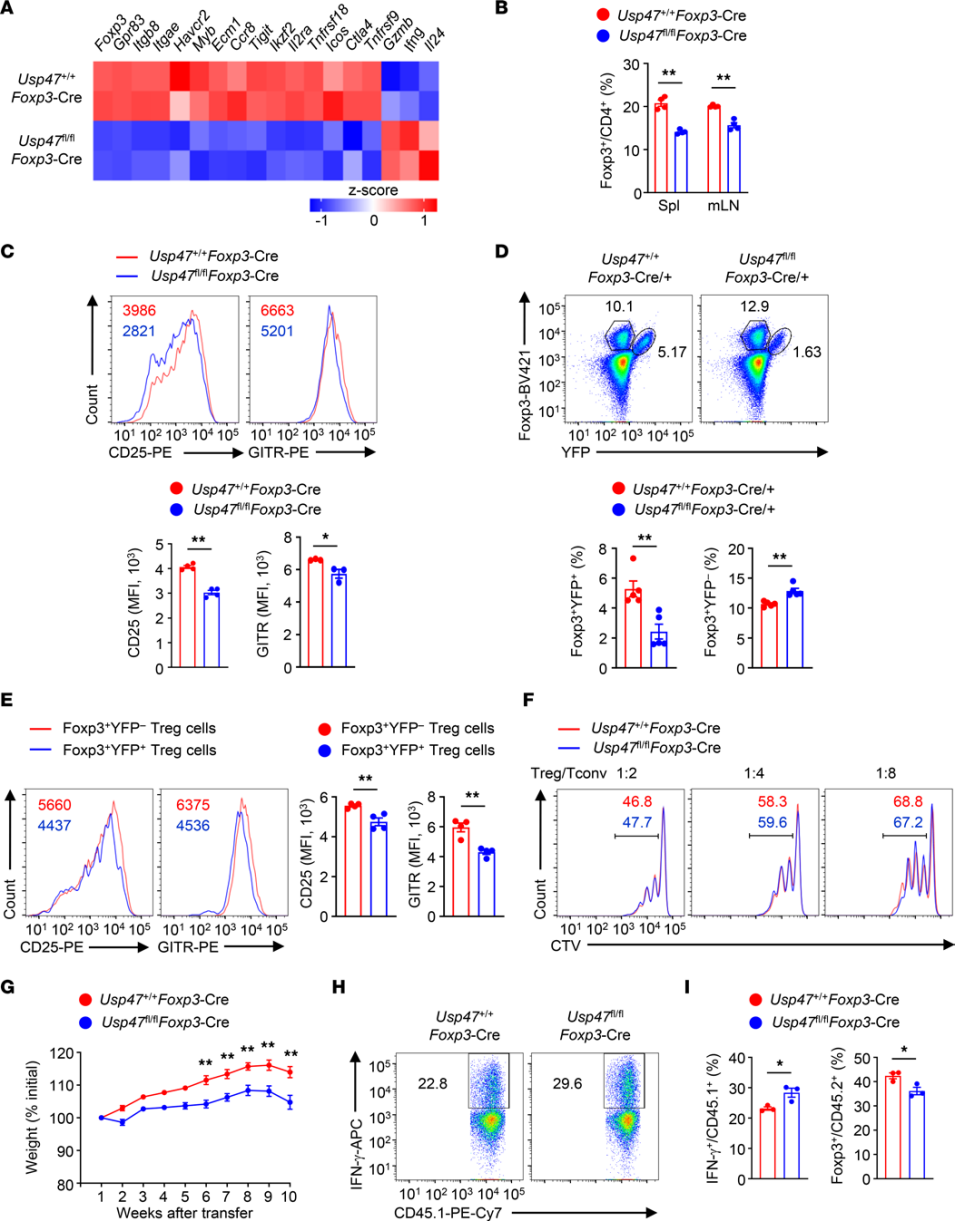
USP47 maintains Treg homeostasis and function in vivo. (**A**) Heatmap of downregulated or upregulated genes associated with Tregs or effector T cells in *Usp47^fl/fl^Foxp3*-Cre Tregs relative to those in *Usp47^+/+^Foxp3*-Cre Tregs stimulated with anti-CD3 and anti-CD28 for 24 hours. (**B**) Flow cytometric analysis of Foxp3^+^CD4^+^ Tregs from spleen (Spl) and mLNs of 3-month-old *Usp47^+/+^Foxp3*-Cre and *Usp47^fl/fl^Foxp3*-Cre mice (*n* = 4). (**C**) Flow cytometric analysis of expression levels of CD25 and GITR on CD4^+^Foxp3^+^ Tregs of 3-month old *Usp47^+/+^Foxp3*-Cre and *Usp47^fl/fl^Foxp3*-Cre mice (*n* = 4 for CD25 and *n* = 3 for GITR). The numbers above the graphs indicate the MFI. (**D**) Flow cytometric analysis of the proportions of Foxp3^+^YFP^+^ and Foxp3^+^YFP^–^ Tregs in the spleen from 3-month-old *Usp47^+/+^Foxp3*-Cre/+ and *Usp47^fl/fl^Foxp3*-Cre/+ female mice (*n* = 5). (**E**) Flow cytometric analysis of CD25 and GITR expression levels on Foxp3^+^YFP^+^ and Foxp3^+^YFP^–^ Tregs in the spleen from 3-month-old *Usp47^fl/fl^Foxp3*-Cre/+ female mice (*n* = 4). (**F**) Flow cytometric analysis of T cell proliferation. *Usp47^+/+^Foxp3*-Cre or *Usp47^fl/fl^Foxp3*-Cre Tregs were cocultured with naive CD4^+^ (conventional [Tconv]) T cells labeled with CellTrace Violet (CTV) at the indicated ratios and were activated with Dynabeads Mouse T-activator CD3/CD28. CTV signal was assessed after 3 days. (**G**–**I**) WT (CD45.1^+^) naive CD45RB^hi^CD4^+^ T cells together with Tregs isolated from 8-week-old *Usp47^+/+^Foxp3*-Cre or *Usp47^fl/fl^Foxp3*-Cre mice were adoptively transferred into 8-week-old RAG-1–deficient mice. Body weight is relative to initial weight (**G**; *n* = 5). Flow cytometric analysis of IFN-γ–producing CD45.1^+^ T cells and Foxp3^+^CD45.2^+^ T cells in the mLNs (**H** and **I**; *n* = 3). Representative data are shown from 3 (**B**–**E**) and 2 (**F**–**I**) independent experiments. Data are presented as mean ± SEM. *P < 0.05; **P < 0.01. Two-tailed Student’s *t* test (**B**–**E**, **G**, and **I**).

Performing a transcriptome analysis, we found that USP47-deficient Tregs failed to induce the expression of many Treg signature genes, including *Foxp3*, *Il2ra*, and *Tnfrsf18* (Figure 4A). Three-month-old *Usp47^fl/fl^Foxp3*-Cre mice displayed a profound reduction in the frequency of Foxp3^+^ Tregs (Figure 4B). In addition, Foxp3^+^ USP47-deficient Tregs exhibited lower expression of the Treg markers CD25 and GITR (Figure 4C). We further analyzed the phenotype of Tregs from female *Usp47^fl/fl^Foxp3*-Cre/+ mice, in which approximately one-half of the Tregs (Foxp3^+^YFP^–^) expressed sufficient USP47, while the other one-half of the Tregs (Foxp3^+^YFP^+^) were deficient in USP47 expression due to the random X chromosome inactivation (38). Notably, the percentage of Foxp3^+^YFP^+^ Tregs in female *Usp47^fl/fl^Foxp3*-Cre/+ mice was significantly lower than that in female *Usp47^+/+^Foxp3*-Cre/+ mice, whereas the percentage of Foxp3^+^YFP^–^ Tregs in female *Usp47^fl/fl^Foxp3*-Cre/+ mice was higher than that in female *Usp47^+/+^Foxp3*-Cre/+ mice (Figure 4D). Moreover, Foxp3^+^YFP^+^ Tregs exhibited lower expression of CD25 and GITR than did Foxp3^+^YFP^–^ Tregs from female *Usp47^fl/fl^Foxp3*-Cre/+ mice (Figure 4E). Furthermore, Foxp3^+^YFP^+^ Tregs exhibited lower mRNA levels of *Foxp3*, *Il2ra*, *Tnfrsf18*, and *Ctla4* than did Foxp3^+^YFP^–^ Tregs from female *Usp47^fl/fl^Foxp3*-Cre/+ mice (Supplemental Figure 3A). These data suggest that USP47 sustains Treg homeostasis in vivo in an intrinsic manner.

We measured Treg function by using an in vitro suppression assay and found that USP47-deficient Tregs exhibited normal suppressive function in vitro (Figure 4F). We further established a T cell transfer–induced colitis model to test the effect of USP47 on Treg function in vivo. In this model, *Rag1*-KO mice received CD45.1^+^CD4^+^CD45RB^hi^ T cells and CD45.2^+^ Tregs from *Usp47^+/+^Foxp3*-Cre or *Usp47^fl/fl^Foxp3*-Cre mice. The cotransfer of USP47-deficient Tregs with CD45RB^hi^CD4^+^ T cells resulted in gradual weight loss and a greater frequency of IFN-γ–producing effector CD4^+^ T cells (Figure 4, G–I). Importantly, the fraction of Foxp3^+^ cells in mice implanted with USP47-deficient Tregs and CD45RB^hi^CD4^+^ T cells was decreased compared with that in mice implanted with WT Tregs and CD45RB^hi^CD4^+^ T cells (Figure 4I). These data suggest that USP47 is essential for Treg homeostasis and function in vivo.

### USP47 restricts c-Myc–dependent Treg hyperglycolysis.

**Figure 5:**
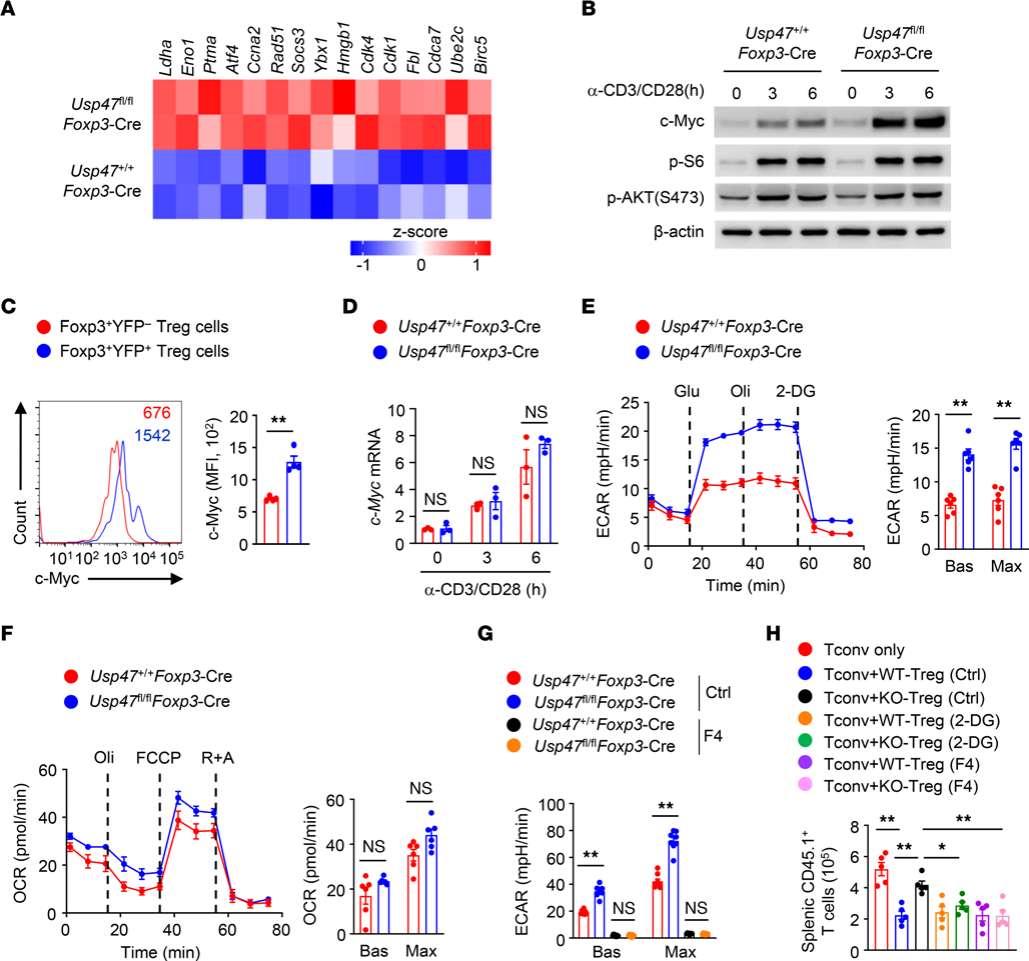
USP47 restricts c-Myc–dependent Treg hyperglycolysis. (**A**) Heatmap of c-Myc–associated genes in Tregs stimulated with anti-CD3 and anti-CD28 for 24 hours. (**B**) Immunoblot analysis of Tregs stimulated with anti-CD3 and anti-CD28 antibodies for 0, 3, or 6 hours. (**C**) c-Myc expression levels in Foxp3^+^YFP^+^ and Foxp3^+^YFP^–^ Tregs in the spleen from 3-month-old *Usp47^fl/fl^Foxp3*-Cre/+ female mice stimulated with anti-CD3 and anti-CD28 antibodies for 3 hours (*n* = 4). (**D**) qRT-PCR analysis of *c-Myc* mRNA levels in Tregs stimulated with anti-CD3 and anti-CD28 antibodies for 0, 3, or 6 hours (*n* = 3). (**E**) Extracellular acidification rates (ECAR) of Tregs stimulated with anti-CD3 and anti-CD28 antibodies for 3 hours under basal conditions (Bas) or at maximum (Max) with the addition of glucose (Glu), oligomycin (Oli), and 2-deoxy-d-glucose (2-DG) (*n* = 6). (**F**) Oxygen consumption rates (OCR) of Tregs stimulated with anti-CD3 and anti-CD28 antibodies for 3 hours under basal conditions (Bas) or at maximum (Max) with the addition of oligomycin, the mitochondrial uncoupler FCCP, and rotenone plus antimycin A (R+A) (*n* = 6). (**G**) ECAR of Tregs stimulated with anti-CD3 and anti-CD28 antibodies for 3 hours in the presence of 100 μM 10058-F4 (F4) (*n* = 8). Ctrl, DMSO. (**H**) CD45.1^+^ conventional CD4^+^ T cells (Tconv) were injected i.v. into *Rag1*-KO mice alone or with an equal number of CD45.2^+^ *Usp47^+/+^Foxp3*-Cre (WT) or *Usp47^fl/fl^Foxp3*-Cre (KO) Tregs pretreated with anti-CD3 and anti-CD28 antibodies plus 2 mM 2-DG or 100 μM 10058-F4 (F4) for 6 hours. Day 7 after cell transfer, cells were isolated from spleen (*n* = 5). Data shown are representative of 3 (**B**–**H**) independent experiments and are presented as mean ± SEM. Two-tailed Student’s *t* test (**C**–**F**) or 1 -way ANOVA (**G** and **H**). *P < 0.05; **P < 0.01.

c-Myc–mediated cellular metabolism controls Treg homeostasis and function (15). Interestingly, the expression of c-Myc–associated genes was upregulated in USP47-deficient Tregs (Figure 5A). Indeed, the protein level of c-Myc was higher in activated USP47-deficient Tregs than in activated WT Tregs (Figure 5, B and C), although USP47 deficiency did not enhance the induction of *c-Myc* mRNA (Figure 5D). Given that T cell receptor (TCR) stimulation activates the mTOR signaling pathway, causing c-Myc expression in Tregs (11), we sought to determine whether USP47 regulates TCR-induced mTOR activation. However, the activation of mTORC1 or mTORC2 signaling in USP47-deficient Tregs was not altered (Figure 5B and Supplemental Figure 3B), suggesting that USP47 was dispensable for the mTOR signaling pathway in the Tregs. Since c-Myc is a crucial regulator of Treg metabolism (11, 17), we next examined the involvement of Treg metabolic programs. Interestingly, USP47 deficiency increased the glycolytic rates but not the oxidative phosphorylation rates of Tregs (Figure 5, E and F). To elucidate the importance of c-Myc in Treg hyperglycolysis, we stimulated WT and USP47-deficient Tregs in the presence or absence of the c-Myc inhibitor 10058-F4. The expression of glycolysis-related genes was upregulated in USP47-deficient Tregs (Supplemental Figure 3C). After the addition of 10058-F4, the expression of glycolysis-related genes was greatly diminished in USP47-deficient Tregs (Supplemental Figure 3C). Moreover, pharmacological inhibition of c-Myc activity limited hyperglycolysis of USP47-deficient Tregs (Figure 5G). To define the dependence of defective Treg function on hyperglycolysis, purified CD45.1^+^ conventional CD4^+^ T cells were adoptively transferred into *Rag1*-KO mice alone or with CD45.2^+^ WT or USP47-deficient Tregs pretreated with a glycolysis inhibitor, 2-deoxy-d-glucose (2-DG). Indeed, 2-DG treatment restored the suppressive functions of USP47-deficient Tregs (Figure 5H). Importantly, specifically attenuating c-Myc activity restored the suppressive functions of USP47-deficient Tregs (Figure 5H). Therefore, USP47 inhibits excessive c-Myc activity and hyperglycolysis in Tregs.

### USP47 decreases m^6^A-based c-Myc translation efficiency in Tregs.

**Figure 6:**
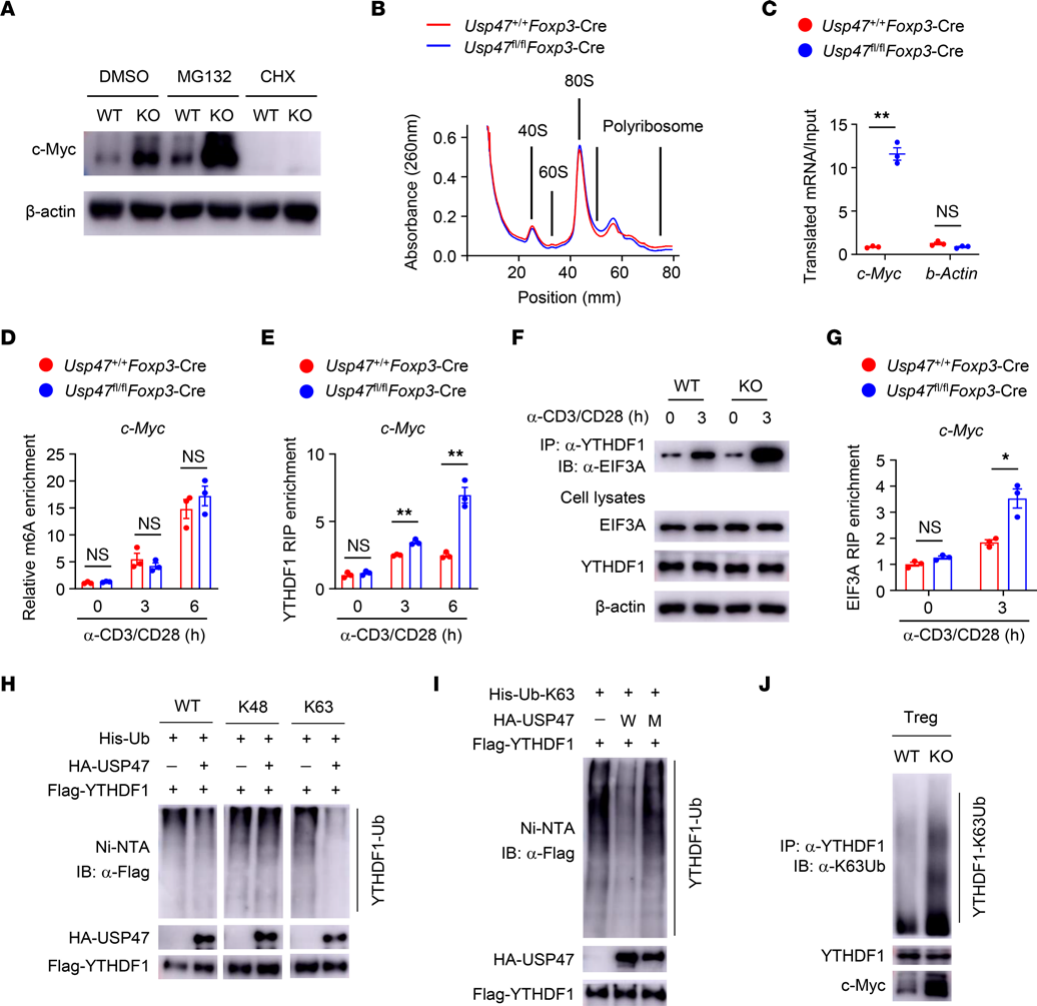
USP47 decreases m^6^A-based c-Myc translation efficiency in Tregs. (**A**) Immunoblot analysis of Tregs stimulated with anti-CD3 and anti-CD28 antibodies plus DMSO, MG132, or cycloheximide (CHX) for 1.5 hours. (**B**) Representative trace of ribosome extract prepared from stimulated Tregs in the presence of CHX. (**C**) Ribosome occupancy of *c-Myc* and control (*b-actin*) mRNA was measured by real-time PCR as the relative expression ratio of polyribosome mRNAs to the input mRNAs after sucrose gradient fraction of polyribosomes (*n* = 3). (**D**) m^6^A-RIP-qRT-PCR analysis of m^6^A levels of *c-Myc* mRNA in Tregs stimulated with anti-CD3 and anti-CD28 antibodies for 0, 3, or 6 hours (*n* = 3). (**E**) RIP analysis of the interaction of YTHDF1 with *c-Myc* mRNA in Tregs stimulated with anti-CD3 and anti-CD28 antibodies for 0, 3, or 6 hours. The enrichment was measured by qRT-PCR and normalized to input (*n* = 3). (**F**) Lysates from *Usp47^+/+^Foxp3*-Cre (WT) and *Usp47*^fl/fl^*Foxp3*-Cre (KO) Tregs stimulated with anti-CD3 and anti-CD28 antibodies for 0 or 3 hours were subjected to immunoprecipitation with anti-YTHDF1 antibodies. (**G**) RIP analysis of the interaction of eIF3A with *c-Myc* mRNA in Tregs stimulated with anti-CD3 and anti-CD28 antibodies for 0 or 3 hours (*n* = 3). (**H**) Immunoblot analysis of YTHDF1 ubiquitination (Ub) in HEK293T cells transfected with the indicated vectors. (**I**) Immunoblot analysis of YTHDF1 ubiquitination in HEK293T cells transfected with the indicated vectors. W, WT USP47; M, mutant USP47 C197S lacking the E3 ligase activity. (**J**) Immunoblot analysis of YTHDF1 K63-linked ubiquitination in *Usp47^+/+^Foxp3*-Cre (WT) and *Usp47^fl/fl^Foxp3*-Cre (KO) Tregs stimulated with anti-CD3 and anti-CD28 antibodies for 3 hours. Data shown are representative of 3 independent experiments and are presented as mean ± SEM. *P < 0.05; **P < 0.01. Two-tailed Student’s *t* test (**C**–**E** and **G**).

To determine whether the hyperactivation of c-Myc is due to enhanced stabilization or expression of c-Myc, we treated activated Tregs with the proteasome inhibitor MG132 or the protein synthesis inhibitor cycloheximide (CHX). Blocking protein degradation with MG132 did not eliminate the difference in c-Myc protein levels between USP47-deficient and USP47-sufficient Tregs (Figure 6A). In contrast, treatment with CHX substantially lowered the protein level of c-Myc and eliminated the difference between USP47-deficient and USP47-sufficient Tregs (Figure 6A). Moreover, there were no significant differences in the c-Myc protein half-life between WT and USP47-deficient Tregs (Supplemental Figure 4A), suggesting that USP47 does not affect c-Myc protein stability. Because USP47 deficiency did not enhance the induction of *c-Myc* mRNA (Figure 5D), we investigated whether USP47 regulates c-Myc translation. To directly test the possibility that USP47 affects c-Myc translation, polysome profiling was analyzed. Ribosomes in the Treg lysate were divided into small (40S) and large (60S) ribosomal subunits, monosomes (80S), and polysomes (Figure 6B). We further performed polyribosome real-time PCR experiments to quantify the ribosome occupancy of *c-Myc* mRNAs. Notably, we found a dramatic increase in the accumulation of polyribosomes on *c-Myc* mRNAs but not on *b-actin* transcripts upon USP47 depletion (Figure 6C). These data suggest that USP47 is essential for efficient *c-Myc* mRNA translation in Tregs. m^6^A methylation promotes translation efficiency, and the m^6^A reader protein YTHDF1 preferentially binds to the methylated mRNA to mediate the translation of m^6^A-modified mRNAs (39). Gene-specific m^6^A assays revealed TCR-induced m^6^A enrichment of *c-Myc* mRNA in Tregs (Figure 6D). USP47 ablation exerted no effect on the m^6^A modification status of *c-Myc* mRNA (Figure 6D). Interestingly, a TCR-induced interaction between YTHDF1 and *c-Myc* mRNA was observed (Figure 6E). Importantly, depletion of USP47 resulted in increased interaction between YTHDF1 and *c-Myc* mRNA in Tregs (Figure 6E). YTHDF1 has been reported to bind m^6^A-modified mRNAs and increases translational output through its interactions with initiation factors, such as eIF3A and eIF3B (39). Indeed, the association of YTHDF1 with eIF3A was induced after TCR stimulation, and this interaction was facilitated in USP47-deficient Tregs (Figure 6F). Moreover, an augmented interaction between eIF3A and *c-Myc* mRNA was detected in USP47-deficient Tregs (Figure 6G). These results suggest that loss of USP47 promotes YTHDF1-dependent m^6^A-based c-Myc translation efficiency in Tregs.

USP47 is a DUB that mediates protein deubiquitination and regulates protein-protein interactions (40–42). Our immunoblot analyses revealed that overexpression of USP47 with WT ubiquitin or K63-ubiquitin (K63), but not with K48-ubiquitin, inhibited YTHDF1 ubiquitination in HEK293T cells (Figure 6, H and I). In contrast, catalytically inactive USP47 did not deubiquitinate YTHDF1 in HEK293T cells (Figure 6I). Moreover, abundant K63-linked ubiquitination (K63-Ub) of YTHDF1 was observed in activated USP47-deficient Tregs (Figure 6J). Using the PhosphoSitePlus posttranslational modification database, we predicted ubiquitination motifs on YTHDF1 at residues K370, K372, and K500. Then, we constructed plasmids expressing WT or mutant YTHDF1, in which the predicted lysine (K) residues were replaced by arginine (R) to block ubiquitination. YTHDF1 was at least conjugated by K63-linked ubiquitination at residue K500 (Supplemental Figure 4B). Indeed, K63-linked ubiquitination at residue K500 did not affect the stability of YTHDF1 (Supplemental Figure 4C). Notably, K63-linked ubiquitination at residue K500 of YTHDF1 promoted YTHDF1-eIF3A interaction (Supplemental Figure 4C). These data indicate that USP47 prevents YTHDF1 ubiquitination and then attenuates the association of YTHDF1 with the translation initiation machinery in the Tregs.

### YTHDF1 involves USP47-mediated Treg metabolism and homeostasis.

**Figure 7:**
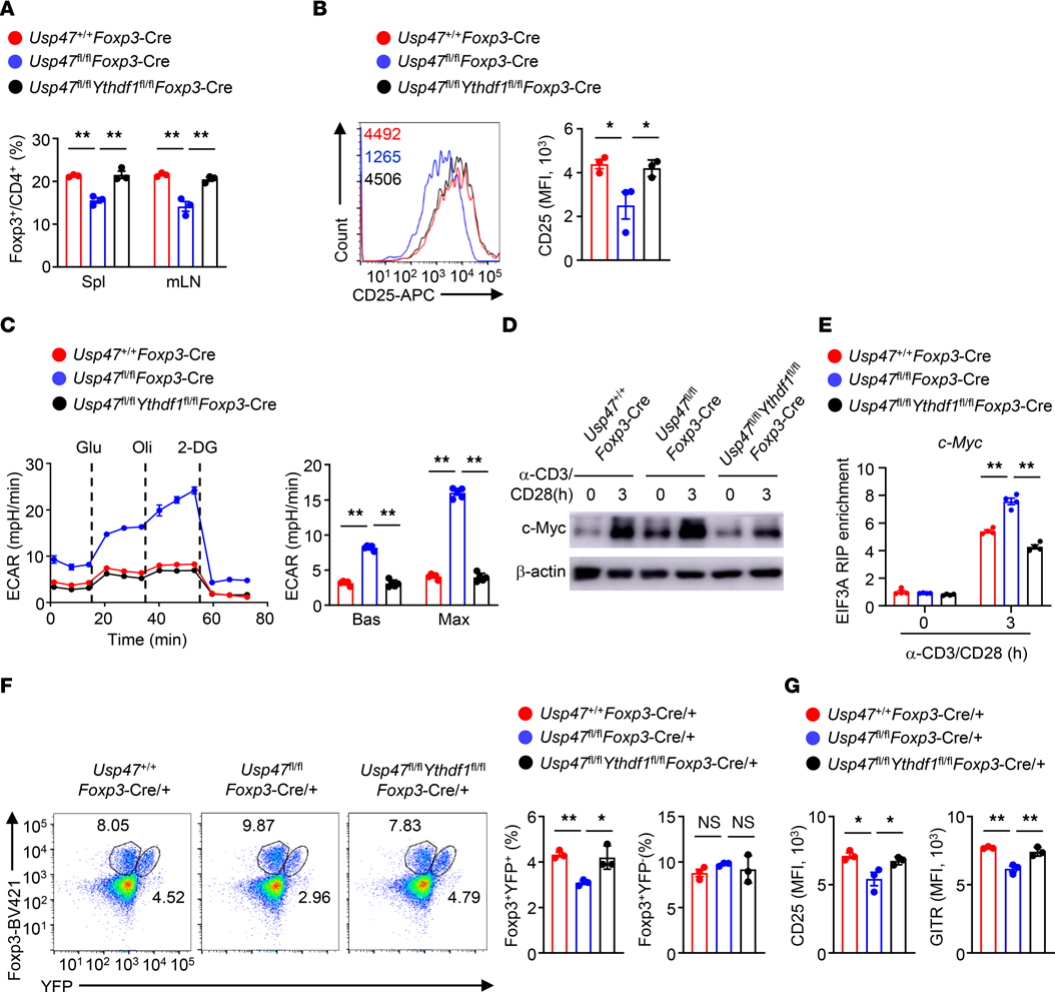
YTHDF1 involves USP47-mediated Treg metabolism and homeostasis. (**A**) Flow cytometric analysis of the proportions of CD4^+^Foxp3^+^ Tregs in the spleen (Spl) and mLNs from 3-month-old *Usp47^+/+^Foxp3*-Cre, *Usp47^fl/fl^Foxp3*-Cre, and *Usp47^fl/fl^Ythdf1^fl/fl^Foxp3*-Cre mice (*n* = 3). (**B**) Flow cytometric analysis of CD25 expression levels on Foxp3^+^YFP^+^ Tregs in the spleen from 3-month-old *Usp47^+/+^Foxp3*-Cre, *Usp47^fl/fl^Foxp3*-Cre, and *Usp47^fl/fl^Ythdf1^fl/fl^Foxp3*-Cre mice (*n* = 3). (**C**) ECAR of *Usp47^+/+^Foxp3*-Cre, *Usp47^fl/fl^Foxp3*-Cre, and *Usp47^fl/fl^Ythdf1^fl/fl^Foxp3*-Cre Tregs stimulated with anti-CD3 and anti-CD28 antibodies for 3 hours under basal conditions (Bas) or at maximum (Max) with the addition of glucose (Glu), oligomycin (Oli), and 2-DG (*n* = 5). (**D**) Immunoblot analysis of c-Myc expression in Tregs from *Usp47^+/+^Foxp3*-Cre, *Usp47^fl/fl^Foxp3*-Cre, and *Usp47^fl/fl^Ythdf1^fl/fl^Foxp3*-Cre mice stimulated with anti-CD3 and anti-CD28 antibodies for 0 or 3 hours. (**E**) RIP analysis of the interaction of eIF3A with *c-Myc* mRNA in *Usp47^+/+^Foxp3*-Cre, *Usp47^fl/fl^Foxp3*-Cre, and *Usp47^fl/fl^Ythdf1^fl/fl^Foxp3*-Cre Tregs stimulated with anti-CD3 and anti-CD28 antibodies for 0 or 3 hours. The enrichment was measured by qRT-PCR and normalized to input (*n* = 4). (**F**) Flow cytometric analysis of the proportions of Foxp3^+^YFP^+^ and Foxp3^+^YFP^–^ Tregs in the spleen from 3-month-old *Usp47^+/+^Foxp3*-Cre/+, *Usp47^fl/fl^Foxp3*-Cre/+, and *Usp47^fl/fl^Ythdf1^fl/fl^Foxp3*-Cre/+ female mice (*n* = 3). (**G**) Flow cytometric analysis of CD25 and GITR expression levels on Foxp3^+^YFP^+^ Tregs in the spleen from 3-month-old *Usp47^+/+^Foxp3*-Cre/+, *Usp47^fl/fl^Foxp3*-Cre/+, and *Usp47^fl/fl^Ythdf1^fl/fl^Foxp3*-Cre/+ female mice (*n* = 3). Data shown are representative of 3 independent experiments and are presented as mean ± SEM. Statistical analysis was performed using 1-way ANOVA (**A**–**C** and **E**–**G**). *P < 0.05; **P < 0.01.

To verify the importance of YTHDF1 activity in USP47-mediated Treg homeostasis and glycolytic metabolism, we generated *Ythdf1^fl/fl^Foxp3*-Cre mice (Supplemental Figure 5A). Six-week-old *Ythdf1^fl/fl^Foxp3*-Cre mice did not display obvious abnormalities in T cell development or Treg proportion (Supplemental Figure 5, B and C). Notably, YTHDF1 deficiency restored the expression of Foxp3 and CD25 that had been downregulated in the Tregs of 3-month-old *Usp47^fl/fl^Foxp3*-Cre mice (Figure 7, A and B). Compared with Tregs from *Usp47^fl/fl^Foxp3*-Cre mice, Tregs from *Usp47^fl/fl^Ythdf1^fl/fl^Foxp3*-Cre mice showed attenuated glycolytic rates, decreased protein expression of c-Myc, and impaired interactions between eIF3A and *c-Myc* mRNA (Figure 7, C–E). We further analyzed the phenotype of Tregs from 3-month-old *Usp47^fl/fl^Foxp3*-Cre/+ female mice and *Usp47^fl/fl^Ythdf1^fl/fl^Foxp3*-Cre/+ female mice. Although the *Usp47^fl/fl^Foxp3*-Cre/+ female mice and *Usp47^fl/fl^Ythdf1^fl/fl^Foxp3*-Cre/+ female mice exhibited similar proportions of Foxp3^+^YFP^–^ Tregs, the percentage of Foxp3^+^YFP^+^ Tregs in the *Usp47^fl/fl^Ythdf1^fl/fl^Foxp3*-Cre/+ female mice was higher than that in the *Usp47^fl/fl^Foxp3*-Cre/+ female mice (Figure 7F). Moreover, Foxp3^+^YFP^+^ Tregs from *Usp47^fl/fl^Ythdf1^fl/fl^Foxp3*-Cre/+ female mice showed higher levels of CD25 and GITR than Foxp3^+^YFP^+^ Tregs from *Usp47^fl/fl^Foxp3*-Cre/+ female mice (Figure 7G). Therefore, USP47 acts in a YTHDF1-dependent manner to inhibit m^6^A-dependent c-Myc translation and maintain Treg metabolism and homeostasis.

### YTHDF1 deletion restores the suppressive functions of USP47-deficient Tregs.

**Figure 8:**
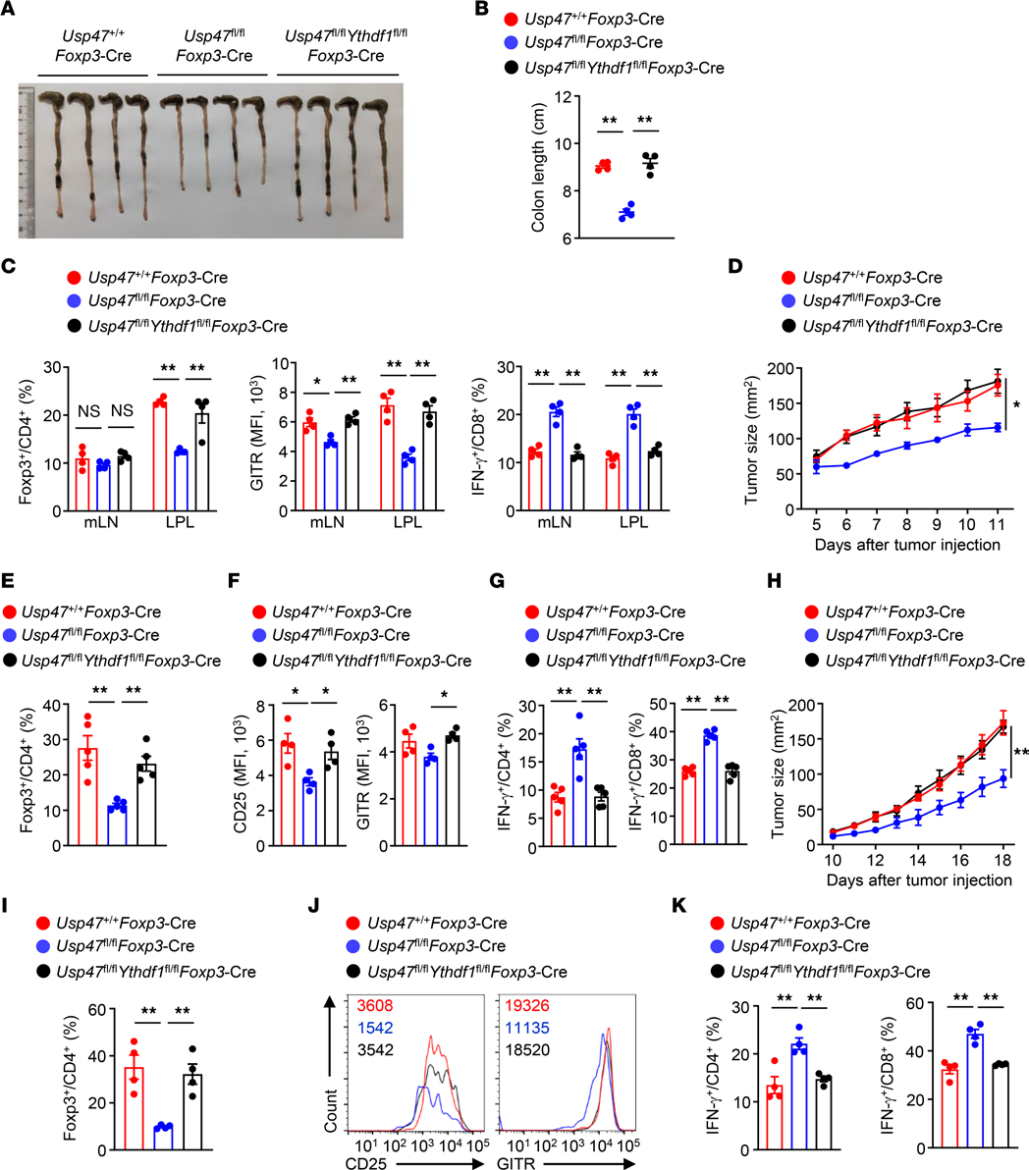
YTHDF1 deletion restores the suppressive functions of USP47-deficient Tregs. (**A**–**C**) Six-week-old *Usp47^+/+^Foxp3*-Cre, *Usp47^fl/fl^Foxp3*-Cre, and *Usp47^fl/fl^Ythdf1^fl/fl^Foxp3*-Cre mice were provided with drinking water containing 2.5% DSS for 7 days, followed by regular water. Mice were killed at day 9. They were analyzed for colon length (**A** and **B**; *n* = 4). Flow cytometric analysis of Foxp3^+^CD4^+^ cells, GITR of Foxp3^+^CD4^+^ cells, and IFN-γ^+^CD8^+^ T cells in the mLNs and lamina propria lymphocytes (LPL) from mice after DSS-induced colitis (**C**; *n* = 4). (**D**–**G**) Tumor growth (**D**; *n* = 5), Treg percentage (**E**; *n* = 5), CD25 and GITR expression in Foxp3^+^ Tregs (**F**; *n* = 4), and percentages of IFN-γ–producing CD4^+^ and CD8^+^ T cells (**G**; *n* = 5) in tumors from 6-week-old *Usp47^+/+^Foxp3*-Cre, *Usp47^fl/fl^Foxp3*-Cre, and *Usp47^fl/fl^Ythdf1^fl/fl^Foxp3*-Cre mice injected subcutaneously with MC38 colon cancer cells. (**H**–**K**) Tumor growth (**H**; *n* = 5), Treg percentage (**I**; *n* = 4), CD25 and GITR expression in Foxp3^+^ Tregs (**J**; *n* = 3), and percentages of IFN-γ–producing CD4^+^ and CD8^+^ T cells (**K**; *n* = 4) in tumors from 6-week-old *Usp47^+/+^Foxp3*-Cre, *Usp47^fl/fl^Foxp3*-Cre, and *Usp47^fl/fl^Ythdf1^fl/fl^Foxp3*-Cre mice injected subcutaneously with B16-F10 cancer cells. Data shown are representative of 3 independent experiments and are presented as mean ± SEM. Statistical analysis was performed using 1-way ANOVA (**B**, **C**, **E**–**G**, **I**, and **K**) or 2-way ANOVA (**D** and **H**). *P < 0.05; **P < 0.01.

To confirm the importance of YTHDF1 in the defective suppressive functions of USP47-deficient Tregs, we performed IBD induction and tumor inoculation using 6-week-old WT mice, *Usp47^fl/fl^Foxp3*-Cre mice, and *Usp47^fl/fl^Ythdf1^fl/fl^Foxp3*-Cre mice. Compared with the *Usp47^fl/fl^Foxp3*-Cre mice, the DSS-treated *Usp47^fl/fl^Ythdf1^fl/fl^Foxp3*-Cre mice displayed attenuated colon length shortening (Figure 8, A and B). Moreover, the percentage of Foxp3^+^ Tregs in the lamina propria and the level of GITR in Foxp3^+^ Tregs from DSS-treated *Usp47^fl/fl^Ythdf1^fl/fl^Foxp3*-Cre mice were higher than those from *Usp47^fl/fl^Foxp3*-Cre mice (Figure 8C). In addition, the frequency of IFN-γ–producing CD8^+^ T cells in the DSS-treated *Usp47^fl/fl^Ythdf1^fl/fl^Foxp3*-Cre mice was lower than that in the DSS-treated *Usp47^fl/fl^Foxp3*-Cre mice (Figure 8C). Compared with the *Usp47^fl/fl^Foxp3*-Cre mice, the *Usp47^fl/fl^Ythdf1^fl/fl^Foxp3*-Cre mice exhibited a profound increase in MC38 or B16-F10 tumor size (Figure 8, D and H). Furthermore, the percentages of tumor-infiltrating Foxp3^+^ Tregs and the levels of CD25 and GITR in tumor-infiltrating Foxp3^+^ Tregs from *Usp47^fl/fl^Ythdf1^fl/fl^Foxp3*-Cre mice were higher than those from *Usp47^fl/fl^Foxp3*-Cre mice (Figure 8, E, F, I, and J). In addition, T cells in the tumors of the tumor-bearing *Usp47^fl/fl^Ythdf1^fl/fl^Foxp3*-Cre mice produced lower levels of IFN-γ than those from the tumor-bearing *Usp47^fl/fl^Foxp3*-Cre mice (Figure 8, G and K). These results suggest that YTHDF1 deletion restores the suppressive functions of USP47-deficient Tregs.

## Discussion

In this work, we report a crucial role for USP47-mediated m^6^A-dependent c-Myc translation in Treg metabolic and functional homeostasis. We found that the Treg signature was associated with higher expression levels of USP47 in human CRC and GC tissues. Targeting USP47 in Tregs exacerbated IBD and enhanced antitumor immunity as a result of impaired Treg homeostasis and suppressive function in vivo. RNA sequencing and biochemical and metabolic analysis demonstrated that USP47 deficiency promoted c-Myc protein accumulation and hyperglycolysis, which contributed to defective Treg homeostasis. Mechanistically, USP47 mediated YTHDF1 deubiquitination and in turn attenuated the association of YTHDF1 with the translation initiation machinery to limit m^6^A-based c-Myc translation in Tregs.

Metabolic reprogramming impacts Treg function and homeostasis (43–45). c-Myc acts as a master regulator targeting most of the genes encoding glycolysis-related enzymes in Tregs (46). In this study, in response to TCR engagement, mTOR signaling was activated to trigger c-Myc expression in T cells, which was coupled with elevated glycolytic metabolism. However, an aberrantly high protein expression and activity of c-Myc compromised Treg survival and function. Autophagy promotes Treg functional integrity by inhibiting mTOR-dependent c-Myc expression (11), but how c-Myc translation efficiency in Tregs is regulated remains unknown. The present study revealed that USP47 prevented the ubiquitination of YTHDF1 and thus prevented its associations with eIF3A to suppress m^6^A-based c-Myc translation in Tregs. USP47-deficient Tregs showed accumulated c-Myc and underwent hyperglycolysis. Indeed, YTHDF1 deficiency distinctly compromised the expression of c-Myc expression and restored the level of glycolysis in USP47-deficient Tregs. Our findings suggest that YTHDF1-involved m^6^A modification governs c-Myc translation efficiency and metabolic reprogramming in Tregs.

The deletion of *Ythdf1* specifically in classic DCs has been reported to reduce the translational efficiency of multiple cathepsin transcripts and enhance the cross-presentation of antigen by DCs to increase antigen-specific CD8^+^ T cell antitumor responses (25). However, the potential role played by YTHDF1 in adaptive immune cells remains unclear. Our data show that Treg-derived YTHDF1 inhibited the translation of c-Myc and c-Myc–dependent glycolysis, which was required for the maintenance of Treg homeostasis. YTHDF1-deficient Tregs with defective suppressive function may contribute to the enhanced antitumor immunity in *Ythdf1*-deficient mice. Our results reveal a role for YTHDF1-mediated m^6^A-based c-Myc translation in Treg metabolism and homeostasis and identify YTHDF1 as a critical mediator of tumor-induced immune tolerance and a potential therapeutic target to improve tumor immunotherapy. Nevertheless, it is still unclear whether YTHDF1 regulates T helper 1 (Th1) cell, Th2 cell, Th17 cell, or CD8^+^ T cell differentiation. The functional role of YTHDF1 in different types of T cells thus needs to be determined.

USP47 removes ubiquitin conjugates from diverse substrates, thereby regulating their stability and activity (41, 42). USP47 is a potential oncogenic protein expressed in various tumor types and mediates tumor cell proliferation and tumor progression (41, 42, 47). Targeting USP47 is a promising strategy to overcome tyrosine kinase inhibitor resistance and eradicate leukemia stem/progenitor cells in chronic myelogenous leukemia (41). Although USP47 regulates inflammasome activation and the release of the proinflammatory cytokines in macrophages (40), its potential role in antitumor immunity is still unknown. In our study, we found that USP47 sustained Treg metabolic and functional fitness and suppressed antitumor T cell immune responses. Together with these findings, we assume that targeting USP47 may not only inhibit tumorigenesis but also elicit potent antitumor immune responses, and both outcomes have important clinical applications.

Our data revealed the importance of c-Myc in hyperglycolysis in USP47-deficient Tregs. Elevated phosphorylated AKT (p-AKT) is recognized as a key mechanism to increase glycolysis during T cell activation (48). However, the level of p-AKT in USP47-deficient Tregs was not altered. Therefore, the potential mechanisms of how elevated Myc is functionally important to temper Treg function in a p-AKT–independent manner in USP47-deficient cells remain to be further investigated.

In conclusion, we discovered a previously unknown role for USP47 in Treg homeostasis and function; it directs m^6^A-based c-Myc translation. Our study demonstrates that Treg-derived USP47 inhibits the assembly of the translation initiation machinery to limit c-Myc translation efficiency and the associated glycolytic metabolism in Tregs. These findings advance our understanding of the role of m^6^A modifications in shaping autoimmunity and antitumor immunity.

## Methods

### Human gastric and colorectal cancer samples.

Human gastric cancer (GC) and colorectal cancer (CRC) tissues and peripheral blood samples from patients were collected at Ruijin Hospital, Shanghai, China. Tumor-infiltrating lymphocytes from GC and CRC tissues were enriched by density-gradient centrifugation. Lymphocytes from PBMCs were also enriched by density-gradient centrifugation. Tregs and non-Treg CD4^+^ T cells were isolated using the CD4^+^CD25^+^ Regulatory T Cell Isolation Kit, human (130-091-301, Miltenyi Biotec). CD8^+^ T cells were isolated using CD8 MicroBeads, human (130-045-201, Miltenyi Biotec).

### Mice.

*Usp47*-floxed mice (in C57BL/6 background) were generated at GemPharmatech Co. Ltd. using a *loxP* targeting system. The *Usp47*-floxed mice were crossed with *Foxp3*-YFP-Cre transgenic mice (in C57BL/6 background) from The Jackson Laboratory to obtain age-matched *Usp47^+/+^Foxp3*-Cre and *Usp47^fl/fl^Foxp3*-Cre mice for experiments. B6.SJL mice and RAG-1–deficient mice in C57BL/6 background were purchased from The Jackson Laboratory. The *Ythdf1*-floxed mice (in C57BL/6 background) were generated at the Shanghai Model Organisms Center Inc. and crossed with *Usp47^fl/fl^Foxp3*-Cre mice to produce *Usp47^fl/fl^Ythdf1^fl/fl^Foxp3*-Cre mice.

### Plasmids, antibodies, and reagents.

FLAG-tagged mouse WT, K370R, K372R, or K500R mutant YTHDF1 was cloned into the lentivirus vector pLVX-IRES-ZsGreen1. HA-tagged mouse WT USP47 and USP47 C197S mutant were cloned into the pcDNA3 vector. Myc-tagged mouse eIF3A was cloned into the pcDNA3.1 vector. His-tagged WT ubiquitin and K48 and K63 mutant ubiquitin were cloned into the pcDNA3 vector. Antibodies against c-Myc (D84C12), p-S6 (D57.2.2E), p-AKT (S473) (D9E), Myc-Tag (71D10), and K63 ubiquitin (D7A11) were purchased from Cell Signaling Technology Inc. HRP-conjugated anti-HA antibody (3F10) was from Roche Inc. HRP-conjugated anti-FLAG (M2) antibody and anti–β-actin antibody were from Sigma-Aldrich Inc. Anti-YTHDF1 antibody was from Proteintech Group Inc. Anti-m^6^A antibody was purchased from Synaptic Systems Inc. Anti-eIF3A antibody was from Novus Biologicals. For flow cytometric experiments, fluorochrome-conjugated antibodies against CD4 (RM4-5), CD8 (53-6.7), CD44 (IM7), CD62L (MEL-14), IFN-γ (XMG1.2), IL-17 (eBio17B7), Foxp3 (FJK-16s), CD25 (PC61.5), GITR (DTA-1), CD45.1 (A20), and CD45.2 (clone 104) were purchased from Thermo Fisher Scientific. The Phospho–S6 Ribosomal Protein (Ser235/236) (D57.2.2E) XP Rabbit mAb (APC conjugate) was purchased from Cell Signaling Technology Inc. Ni-NTA Agarose was purchased from QIAGEN Inc. The c-Myc inhibitor 10058-F4 (F3680) and the glycolysis inhibitor 2-DG (D8375) were purchased from Sigma-Aldrich Inc. RNase inhibitor (2313A) was purchased from Takara Inc.

### Cell lines.

MC38 murine colon cancer cell line, B16-F10 melanoma cell line, and HEK293T cell line were originally from ATCC. All the cell lines used in this study have been authenticated before and were tested negative for mycoplasma contamination.

### Treg isolation and stimulation.

Tregs (CD4^+^CD25^+^YFP^+^) were isolated from the spleen and lymph nodes of age- and sex-matched *Usp47^+/+^Foxp3*-Cre and *Usp47^fl/fl^Foxp3*-Cre mice (6–8 weeks old). The purified Tregs were stimulated with plate-bound anti-CD3 (1 μg/mL) and anti-CD28 (1 μg/mL) and cultured in complete RPMI 1640 medium containing 10% FBS, non-essential amino acids, sodium pyruvate, and antibiotics for flow cytometric, Seahorse, immunoprecipitation, immunoblotting, qPCR, or RNA sequencing analysis.

### RNA sequencing analysis.

Tregs (CD4^+^CD25^+^YFP^+^) were freshly isolated from 6-week-old *Usp47^+/+^Foxp3*-Cre and *Usp47^fl/fl^Foxp3*-Cre mice and stimulated with anti-CD3 (1 μg/mL) and anti-CD28 (1 μg/mL) antibodies in the presence of 200 U/mL IL-2 for 24 hours. Total RNA was isolated from stimulated Tregs with TRIzol (Invitrogen) and subjected to RNA sequencing using Illumina Nextseq500 (75 bp paired-end reads). The RNA sequencing data reported in this paper were deposited in the NCBI Sequence Read Archive with BioProject ID PRJNA907139.

### Flow cytometry.

Freshly isolated cells were stained in PBS containing 2% FBS with indicated fluorochrome-conjugated antibodies to detect surface marker expression. Cells were stimulated with 50 ng/mL phorbol 12-myristate 13-acetate (PMA) and 1 μg/mL ionomycin in the presence of monensin for 5 hours to determine intracellular cytokine expression. Cells were stained with fluorochrome-conjugated antibodies for surface markers, permeabilized, and stained with indicated antibodies for intracellular cytokines according to the manufacturer’s instructions (Thermo Fisher Scientific). For staining of p-S6, freshly isolated Tregs were stimulated with plate-bound anti-CD3 and anti-CD28 for 4 hours, fixed in Fix Buffer I (BD Phosflow) at 37°C for 10 minutes, incubated in cold Perm Buffer III (BD Phosflow) for 20 minutes, and subsequently subjected to p-S6 antibody staining and flow cytometric analysis. Foxp3 staining was performed according to the manufacturer’s protocol (Thermo Fisher Scientific). For Foxp3 staining with intact YFP, freshly splenic cells were stained with antibodies for surface markers first, then fixed with 3.7% formaldehyde and permeabilized with 0.2% Triton X-100, followed by staining with Pacific blue–anti-Foxp3 antibody at 4°C for 2 hours.

### Histology.

Colon and lung tissues were obtained from age- and sex-matched 10-month-old *Usp47^+/+^Foxp3*-Cre and *Usp47^fl/fl^Foxp3*-Cre mice or age- and sex-matched dextran sodium sulfate–treated (DSS-treated) *Usp47^+/+^Foxp3*-Cre and *Usp47^fl/fl^Foxp3*-Cre mice, fixed in 10% neutral-buffered formalin, embedded in paraffin, and sectioned for staining with H&E.

### Colitis model.

For the DSS-induced colitis model, age- and sex-matched *Usp47^+/+^Foxp3*-Cre, *Usp47^fl/fl^Foxp3*-Cre, and *Usp47^fl/fl^Ythdf1^fl/fl^Foxp3*-Cre mice were subjected to 1 cycle of 2.5% DSS in sterile drinking water for 7 days followed by normal drinking water until the completion of the experiment. Images of mini-endoscopic colon, colon lengths, and histopathology were measured for each mouse at the end of the study.

### Tumor model.

MC38 murine colon cancer cells were cultured in DMEM containing 10% FBS. B16-F10 melanoma cells were cultured in RPMI 1640 supplemented with 10% FBS. These cancer cells were tested negative for mycoplasma contamination and were authenticated earlier. These cancer cells (5 × 10^5^ per mouse) were subcutaneously injected into age- and sex-matched *Usp47^+/+^Foxp3*-Cre, *Usp47^fl/fl^Foxp3*-Cre, and *Usp47^fl/fl^Ythdf1^fl/fl^Foxp3*-Cre mice. Tumor size was recorded from day 5 after tumor injection and was calculated by length × width. Tumor-bearing mice with a tumor size larger than 225 mm^2^ were euthanized.

### T cell transfer–induced colitis model.

Naive CD4^+^ T cells (CD4^+^CD25^–^CD45RB^hi^) from B6.SJL (CD45.1^+^) mice and Tregs (CD4^+^CD25^+^YFP^+^) from *Usp47^+/+^Foxp3*-Cre or *Usp47^fl/fl^Foxp3*-Cre (CD45.2^+^) mice were purified by flow cytometry. RAG-1–deficient mice were given intravenous injection of *Usp47^+/+^Foxp3*-Cre or *Usp47^fl/fl^Foxp3*-Cre Tregs (2 × 10^5^) together with naive CD4^+^ T cells (4 × 10^5^). Recipient mice were assessed for body weight weekly and were sacrificed 10 weeks after transfer for flow cytometric analysis.

### In vitro suppression assay.

Naive CD4^+^ T cells (CD4^+^CD25^–^CD62L^hi^) were sorted from WT mice, and Tregs (CD4^+^CD25^+^YFP^+^) were sorted from *Usp47^+/+^Foxp3*-Cre or *Usp47^fl/fl^Foxp3*-Cre mice by flow cytometry. Naive CD4^+^ T cells were labeled with 2.5 μM CellTrace Violet (CTV, Invitrogen) at 37°C for 20 minutes, activated with Dynabeads Mouse T-activator CD3/CD28 (Invitrogen), and cocultured with Tregs at different ratios. Three days later, CTV-labeled CD4^+^ T cell proliferation was examined by flow cytometric analysis.

### In vivo suppression assay.

Conventional CD4^+^ T cells (CD4^+^CD25^–^) from B6.SJL (CD45.1^+^) mice and Tregs (CD4^+^CD25^+^) from *Usp47^+/+^Foxp3*-Cre or *Usp47^fl/fl^Foxp3*-Cre (CD45.2^+^) mice were sorted using the CD4^+^CD25^+^ Regulatory T Cell Isolation Kit, mouse (130-091-041, Miltenyi Biotec). Freshly isolated Tregs were pretreated with anti-CD3 and anti-CD28 antibodies plus DMSO, 2 mM 2-DG, or 100 μM 10058-F4 for 6 hours. RAG-1–deficient mice were given intravenous injection of CD45.1^+^ conventional CD4^+^ T cells (1 × 10^6^) alone or together with *Usp47^+/+^Foxp3*-Cre or *Usp47^fl/fl^Foxp3*-Cre CD45.2^+^ Tregs (1 × 10^6^). Recipient mice were sacrificed 7 days after transfer, and splenocytes were isolated for flow cytometric analysis.

### Glycolytic and mitochondrial respiration rate measurement.

Freshly isolated Tregs from *Usp47^+/+^Foxp3*-Cre, *Usp47^fl/fl^Foxp3*-Cre, and *Usp47^fl/fl^Ythdf1^fl/fl^Foxp3*-Cre mice were stimulated with anti-CD3 and anti-CD28 for 3 hours. The stimulated Tregs were seeded at a density of 4 × 10^5^ per well in a 96-well Seahorse assay plate. The extracellular acidification rate and the oxygen consumption rate for each well were measured by a Seahorse XFe96 Extracellular Flux Analyzer (Agilent Technologies). The cells were subjected to the XF Cell Mito or the XF Glycolytic stress test with the treatment of injected compounds: glucose (10 mM), oligomycin (2 μM), 2-DG (50 mM), carbonyl cyanide 4-(trifluoromethoxy)phenylhydrazone (FCCP; 1 μM), rotenone/antimycin A (0.5 μM). The XF Cell Mito and the XF Glycolytic stress test kits were purchased from Agilent Technologies Inc.

### Immunoprecipitation, immunoblot, and ubiquitination assays.

Stimulated Tregs were rinsed with ice-cold PBS and lysed at 4°C for 30 minutes in RIPA buffer (50 mM Tris-HCl, pH 7.5; 135 mM NaCl; 1% NP-40; 10% glycerol; 5% sodium deoxycholate; 1 mM EDTA) containing protease inhibitor (1:100; P8340, Sigma-Aldrich) and 1 mM PMSF. Cell lysates were cleared by centrifugation at 13,523*g* for 10 minutes. For immunoprecipitation (IP), the supernatants were immunoprecipitated with the appropriate antibodies at 4°C overnight, followed by incubation with protein A/G–agarose beads at 4°C for 1 hour. After incubation, the immunoprecipitated proteins were washed 5 times with the lysis buffer. Samples were denatured by the addition of SDS loading buffer and boiling at 100°C for 10 minutes, resolved by 8% or 10% SDS-PAGE, and analyzed by immunoblotting (IB) analysis with indicated antibodies. For ubiquitination assay in Tregs, YTHDF1 was immunoprecipitated from stimulated Tregs, and the ubiquitinated YTHDF1 was detected by IB using ubiquitin antibodies. For ubiquitination assay in HEK293T cells, the expression vectors encoding YTHDF1 or USP47 along with the His-ubiquitin expression vectors were transfected into HEK293T cells, the His-ubiquitin was isolated by Ni-NTA agarose, and the ubiquitinated YTHDF1 was examined by anti-FLAG IB.

### Quantitative PCR.

Total RNA was extracted from Tregs from human PBMC and tumor tissue samples as well as *Usp47^+/+^Foxp3*-Cre and *Usp47^fl/fl^Foxp3*-Cre stimulated Tregs using TRIzol (Invitrogen). NovoScript Plus All-in-one 1st Strand cDNA Synthesis SuperMix (cDNA Purge, Novoprotein Scientific Inc.) was used for cDNA synthesis, and qPCR was run on a 7500 Fast Real-Time PCR System or ViiA7 Real-Time PCR System using Hieff qPCR SYBR Green Master Mix (Low ROX Plus, Yeasen Biotechnology Co., Ltd.) with gene-specific primers. Data were generated with the comparative threshold cycle (ΔCT) method by normalizing to *b-actin* reference gene. Primers used for human samples were as follows: *USP47* forward (F): CTCGACGCTAATTTTGAGCCA; *USP47* reverse (R): CTCTTGGAAGCGGACCTATAAAC; *FOXP3*-F: GTGGCCCGGATGTGAGAAG; *FOXP3*-R: GAGCCCTTGTCGGATGATG; *CTLA4*-F: CATGATGGGGAATGAGTTGACC; *CTLA4*-R: TCAGTCCTTGGATAGTGAGGTTC; *IL2RA*-F: GTGGGGACTGCTCACGTTC; *IL2RA*-R: CCCGCTTTTTATTCTGCGGAA; *TIGIT*-F: TGGTCGCGTTGACTAGAAAGA; *TIGIT*-R: GGGCTCCATTCCTCCTGTC; *TNFRSF9*-F: TTGGATGGAAAGTCTGTGCTTG; *TNFRSF9*-R: AGGAGATGATCTGCGGAGAGT; *TNFRSF18*-F: CAGTCCCAGGGGAAATTCAGT; *TNFRSF18*-R: GTCTGTCCAAGGTTTGCAGTG; *IFNG*-F: TCGGTAACTGACTTGAATGTCCA; *IFNG*-R: TCGCTTCCCTGTTTTAGCTGC; *IL17A*-F: AGATTACTACAACCGATCCACCT; *IL17A*-R: GGGGACAGAGTTCATGTGGTA; *B-ACTIN*-F: CACCATTGGCAATGAGCGGTTC; *B-ACTIN*-R: AGGTCTTTGCGGATGTCCACGT. Primers used for murine samples were as follows: *c-Myc*-F: CCCTATTTCATCTGCGACGAG; *c-Myc*-R: GAGAAGGACGTAGCGACCG; *Foxp3*-F: ACCATTGGTTTACTCGCATGT; *Foxp3*-R: TCCACTCGCACAAAGCACTT; *Il2ra*-F: AACCATAGTACCCAGTTGTCGG; *Il2ra*-R: TCCTAAGCAACGCATATAGACCA; *Tnfrsf18*-F: GCCATGCTGTATGGAGTCTCG; *Tnfrsf18*-R: CCACTTCCGTTCTGAACCTTG; *Ctla4*-F: TTTTGTAGCCCTGCTCACTCT; *Ctla4*-R: CTGAAGGTTGGGTCACCTGTA; *Pkm2*-F: CGCCTGGACATTGACTCTG; *Pkm2*-R: GAAATTCAGCCGAGCCACATT; *Pgk1*-F: ATGTCGCTTTCCAACAAGCTG; *Pgk1*-R: GCTCCATTGTCCAAGCAGAAT; *Eno1*-F: TGCGTCCACTGGCATCTAC; *Eno1*-R: CAGAGCAGGCGCAATAGTTTTA; *Ldha*-F: CAAAGACTACTGTGTAACTGCGA; *Ldha*-R: TGGACTGTACTTGACAATGTTGG; *Tpi1*-F: CGGGGAGAAGCTAGACGAAAG; *Tpi1*-R: ACACAGGTTCATAGGCCAGGA; *Gpi1*-F: TCAAGCTGCGCGAACTTTTTG; *Gpi1*-R: GGTTCTTGGAGTAGTCCACCAG; *b-actin*-F: CGTGAAAAGATGACCCAGATCA; *b-actin*-R: CACAGCCTGGATGGCTACGT.

### Polyribosome real-time qPCR.

Freshly isolated Tregs from *Usp47^+/+^Foxp3*-Cre and *Usp47^fl/fl^Foxp3*-Cre mice were stimulated with anti-CD3 and anti-CD28 for 6 hours and treated with 100 μg/mL cycloheximide (CHX) for 15 minutes. Then 1 × 10^8^ Tregs were lysed in 400 μL polysome extraction buffer (20 mM Tris-HCl, pH 7.5; 100 mM KCl; 5 mM MgCl_2_; 0.5% NP-40; 100 μg/mL CHX) containing protease inhibitor and 200 U/mL RNase inhibitor. After lysis, one-fourth of the cell lysate was used as input RNA; the remainder was loaded into 5%–50% sucrose gradients and separated by ultracentrifugation with a SW41 rotor (Beckman) at 260,800*g* at 4°C for 3 hours. Linear sucrose gradients were prepared using a Gradient Master (BioComp) as indicated by the manufacturer. Distribution of ribosomes on the gradients was recorded and the polyribosome fractions were collected with a BioComp Piston Gradient Fractionator equipped with a Bio-Rad Econo UV Monitor (set at 260 nm). The input RNAs and polyribosome RNAs were then extracted by TRIzol reagent (Invitrogen) and were used for qRT-PCR analysis.

### RNA immunoprecipitation (RIP).

Stimulated Tregs were collected, washed twice with PBS, and resuspended in IP lysis buffer (25 mM Tris-HCl, pH 7.4; 150 mM KCl; 5 mM EDTA; 0.5 mM DTT; 0.5% NP-40) in the presence of protease inhibitor and 200 U/mL RNase inhibitor. After incubation on ice for 30 minutes, the lysates were harvested by centrifugation at 12,000*g* for 10 minutes. Indicated antibodies or IgG were added into the supernatants followed by incubation overnight at 4°C. Protein A/G beads (Bimake) were then added and incubated with the supernatants for 2 hours at 4°C. The immunoprecipitated complex was extensively washed with IP lysis buffer after incubation, and coprecipitated RNAs were extracted by TRIzol reagent (Invitrogen), followed by qPCR analysis.

### m^6^A-RIP-qPCR.

Total RNA was isolated from stimulated Tregs with TRIzol (Invitrogen) and was further purified with the Dynabeads mRNA Purification Kit (Invitrogen) according to the manufacturer’s instructions. The purified polyadenylated mRNA was incubated with anti-m^6^A antibody or rabbit IgG in IP wash buffer (10 mM Tris-HCl, pH 7.4; 150 mM NaCl; 0.1% NP-40) containing 200 U/mL RNase inhibitor at 4°C overnight. Protein A/G beads (Bimake) were then added and incubated for 2 hours at 4°C. After incubation, the immunoprecipitated beads/m^6^A antibody/mRNA complex was extensively washed 3 times with ice-cold IP wash buffer. TRIzol (Invitrogen) was added again to elute coprecipitated RNA, followed by qPCR analysis.

### Statistics.

Statistical analysis was performed using Prism software (version 7.0, GraphPad). Two-tailed unpaired Student’s *t* tests and 1-way or 2-way ANOVA statistical analyses were performed. Data are presented as mean ± SEM, and *n* is indicated in figure legends. *P* values less than 0.05 are considered statistically significant.

### Study approval.

The patient-derived PBMC and human GC and CRC sample collection was approved by the Clinical Research Ethics Committee of Ruijin Hospital and complied with all relevant ethics regulations. Informed consent was obtained from each patient, and the study protocol was approved by the Clinical Research Ethics Committee of Ruijin Hospital (Ethics Committee Reference Number 2022-239) and complied with all relevant ethics regulations. All mice were maintained in specific pathogen–free facilities, and all mouse experiments were in conformity with protocols approved by the Institutional Animal Care and Use Committee of Shanghai Jiao Tong University School of Medicine.

### Data availability.

The RNA sequencing data reported in this paper were deposited in the NCBI Sequence Read Archive with BioProject ID PRJNA907139. Values for all data points are available in the Supporting Data Values file. All other data relevant to the current study are available from the corresponding authors on reasonable request excluding confidential patient identity information.

## Author contributions

AW and HH designed and performed the experiments, prepared the figures, and wrote the manuscript. JHS, XY, RD, YZ, and QH contributed to the performing of the experiments. ZYN, XL, RZ, and QZ supervised the work and wrote the manuscript.

## Supplementary Material

Supplemental data

Supporting data values

## Version 1 10/03/2023

In-Press Preview

## Version 2 12/01/2023

Electronic publication
