# Supplementary material for: USP47 inhibits m6A-dependent c-Myc translation to maintain regulatory T cell metabolic and functional homeostasis
Source: J Clin Invest. 2023 Dec 1;133(23):e169365. doi: 10.1172/JCI169365 (PMC10688989; doi:10.1172/JCI169365)
Supplement: Supplemental data [file jci-133-169365-s011.pdf]

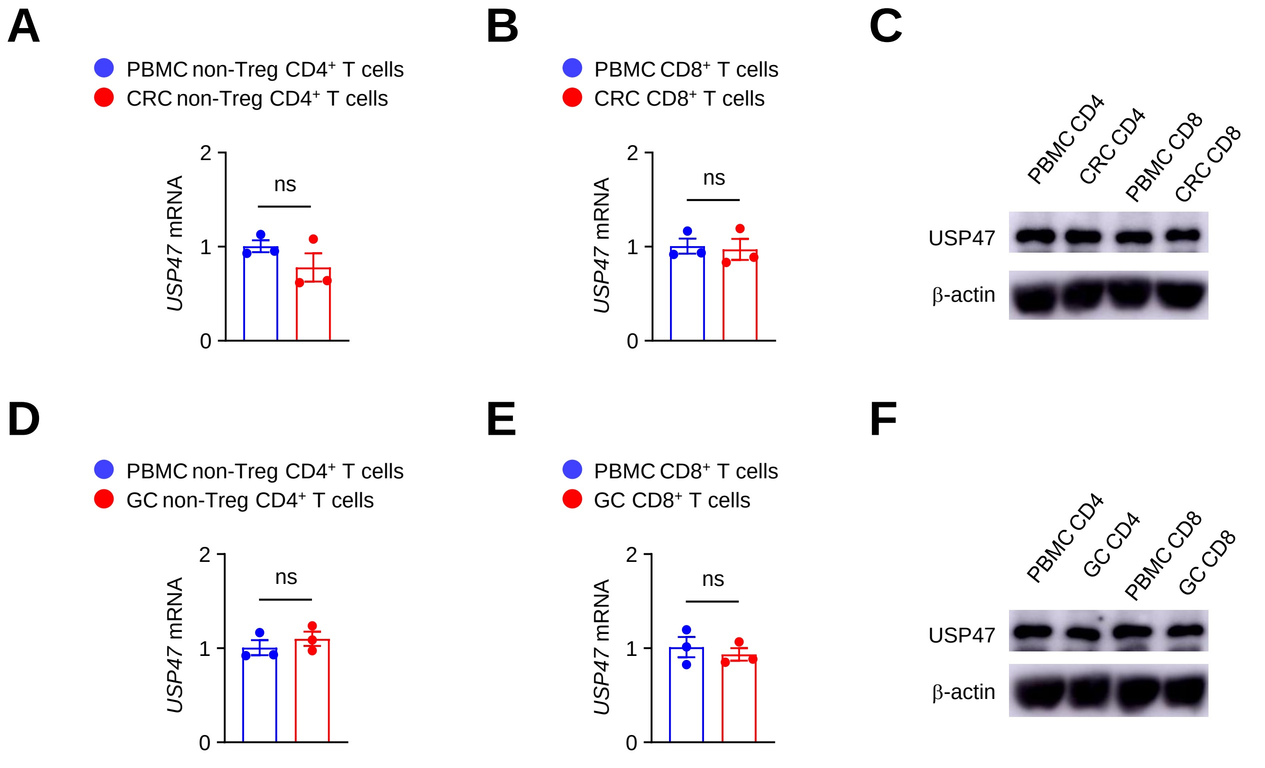

Supplementary Figure 1. USP47 expression in intratumoral non-Treg CD4$^+$ and CD8$^+$ T cells from CRC and GC. (**A** and **B**) qRT-PCR analysis of *USP47* mRNA levels in CD25-negative (non-Treg) CD4$^+$ T cells (**A**; n=3) and CD8$^+$ T cells (**B**; n=3) from peripheral blood mononuclear cells (PBMC) and CRC tissues. (**C**) Immunoblot analysis of USP47 expression in CD25-negative (non-Treg) CD4$^+$ T cells and CD8$^+$ T cells from peripheral blood mononuclear cells (PBMC) and CRC tissues. (**D** and **E**) qRT-PCR analysis of *USP47* mRNA levels in CD25-negative (non-Treg) CD4$^+$ T cells (**D**; n=3) and CD8$^+$ T cells (**E**; n=3) from peripheral blood mononuclear cells (PBMC) and GC tissues. (**F**) Immunoblot analysis of USP47 expression in CD25-negative (non-Treg) CD4$^+$ T cells and CD8$^+$ T cells from peripheral blood mononuclear cells (PBMC) and GC tissues. Data are representative of two independent experiments and are presented as means ±SEM. ns, not statistically significant. Two-tailed Student's *t* test.

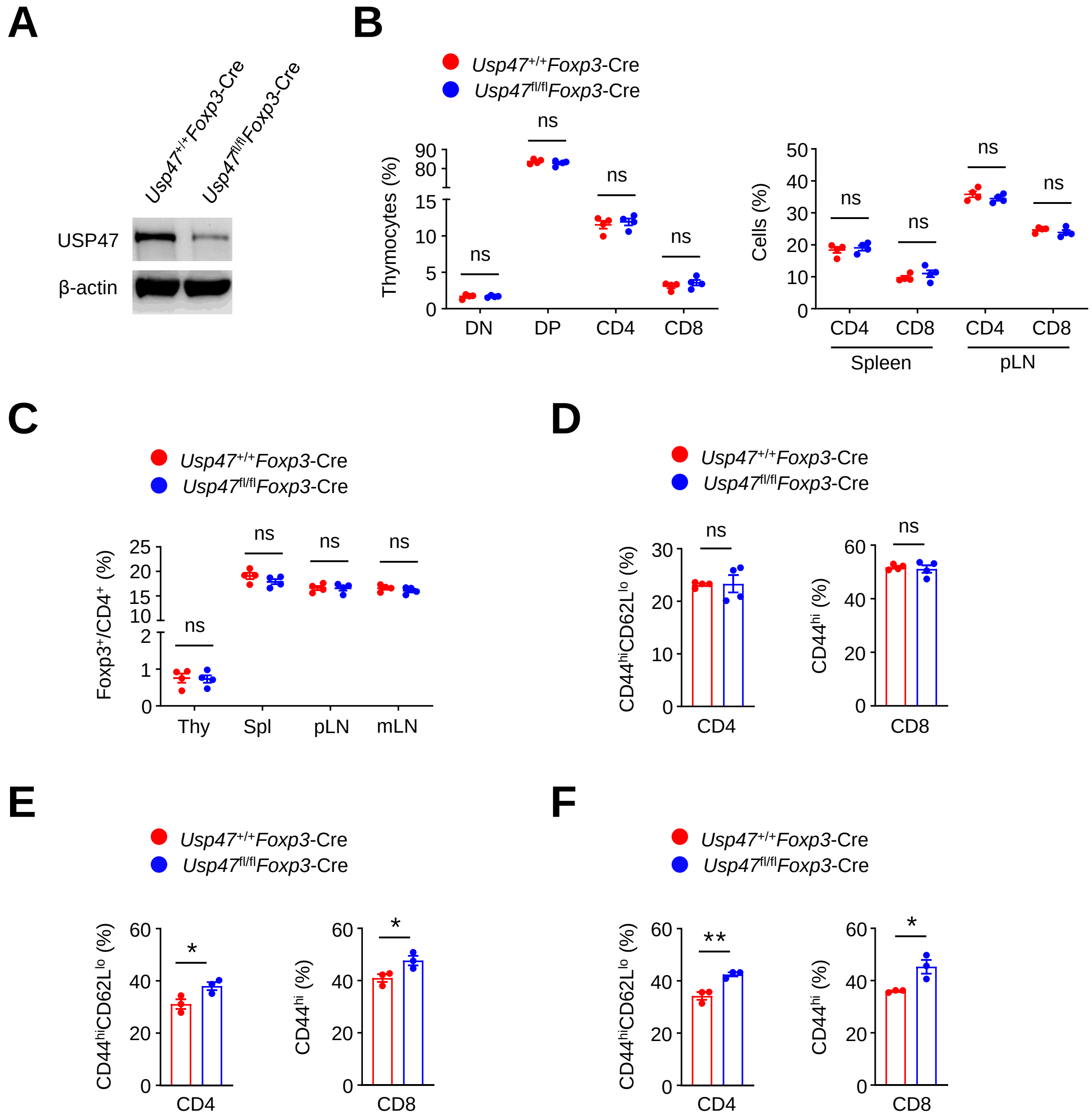

Supplementary Figure 2. T cell development and homeostasis in *Usp47*$^{fl/fl}$*Foxp3*-Cre mice. (A) IB analysis of USP47 using isolated CD4^{+}YFP^{+} cells from *Usp47*$^{+/+}$*Foxp3*-Cre and *Usp47*$^{fl/fl}$*Foxp3*-Cre mice. **(B)** Flow cytometric analysis of the percentage of T cells in the thymus, spleen and peripheral lymph node (pLN) of 6-week-old *Usp47*$^{+/+}$*Foxp3*-Cre and *Usp47*$^{fl/fl}$*Foxp3*-Cre mice (n=4). **(C)** Flow cytometric analysis of the percentage of CD4^{+}Foxp3^{+} T cells in the thymus (Thy), spleen (Spl), pLN and mesenteric lymph node (mLN) of 6-week-old *Usp47*$^{+/+}$*Foxp3*-Cre and *Usp47*$^{fl/fl}$*Foxp3*-Cre mice (n=4). **(D-F)** Flow cytometric analysis of the percentage of CD4^{+}CD44^{hi}CD62L^{lo} and CD8^{+}CD44^{hi} T cells in the spleen of 6-week-old (**D**; n=4), 10-week-old (**E**; n=3), 3-month-old (**F**; n=3) *Usp47*$^{+/+}$*Foxp3*-Cre and *Usp47*$^{fl/fl}$*Foxp3*-Cre mice. Data are representative of three independent experiments and are presented as means ±SEM. ns, not statistically significant; *, $P < 0.05$; **, $P < 0.01$. Two-tailed Student's *t* test.

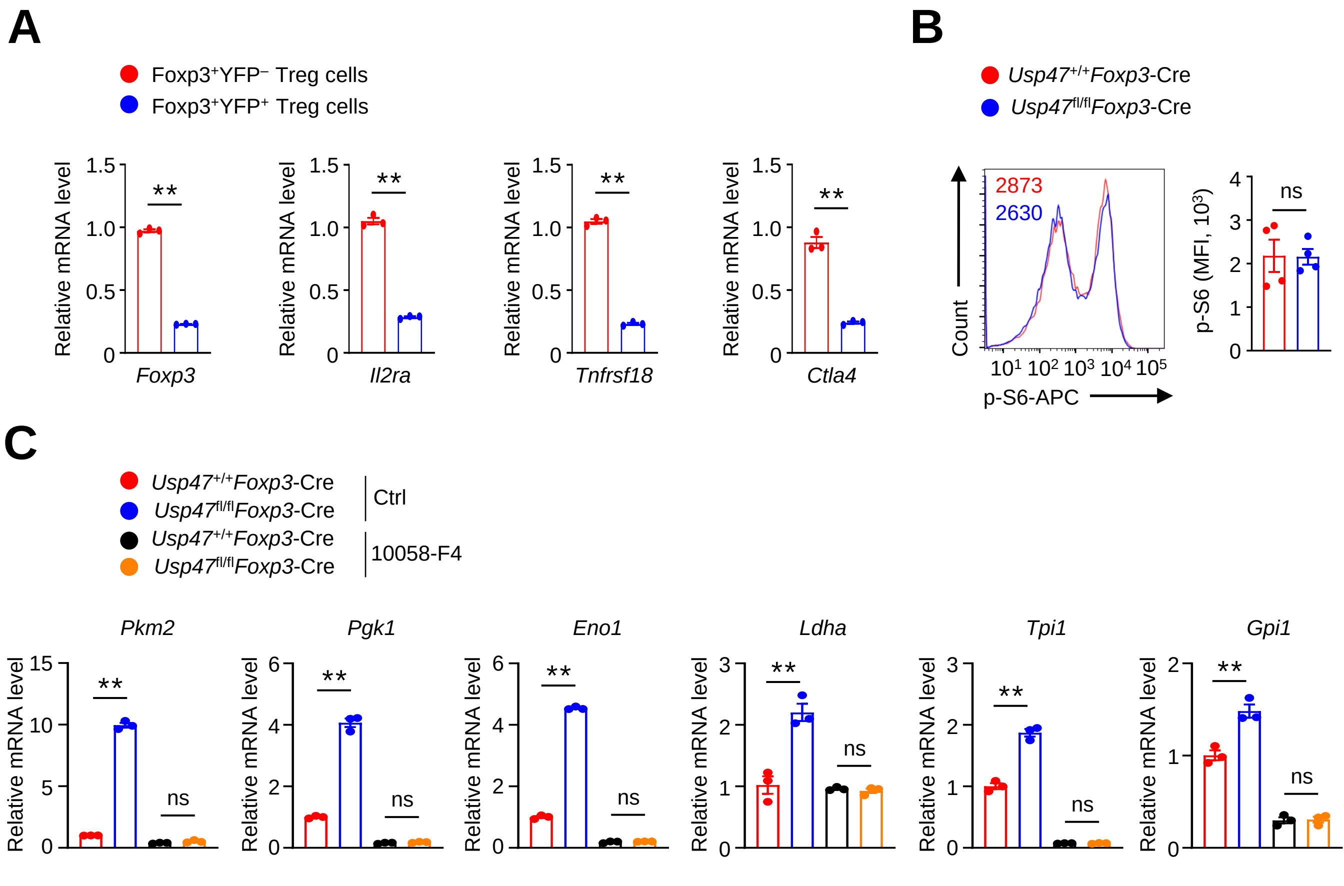

Supplementary Figure 3. Phenotypic characterization of USP47-deficient Treg cells. (A) qRT-PCR analysis of indicated mRNA levels in Foxp3^{+}YFP^{+} and Foxp3^{+}YFP$^{–}$ Treg cells in the spleen from 3-month-old *Usp47*$^{fl/fl}$*Foxp3*-Cre/+ female mice stimulated with anti-CD3 and anti-CD28 antibodies for 3 hours (n=3). **(B)** Flow cytometric analysis of p-S6 expression in Treg cells from *Usp47*$^{+/+}$*Foxp3*-Cre and *Usp47*$^{fl/fl}$*Foxp3*-Cre mice stimulated with anti-CD3 and anti-CD28 antibodies for 4 hours (n=4). **(C)** qRT-PCR analysis of indicated mRNA levels in *Usp47*$^{+/+}$*Foxp3*-Cre and *Usp47*$^{fl/fl}$*Foxp3*-Cre Treg cells stimulated with anti-CD3 and anti-CD28 antibodies for 3 hours in the presence of 100μmol/L 10058-F4 (n=3). Ctrl, DMSO. The data shown are representative of three independent experiments and are presented as mean ± SEM. ns, not statistically significant; **, P < 0.01. Two-tailed Student's *t* test (**A, B**) or one-way ANOVA (**C**).

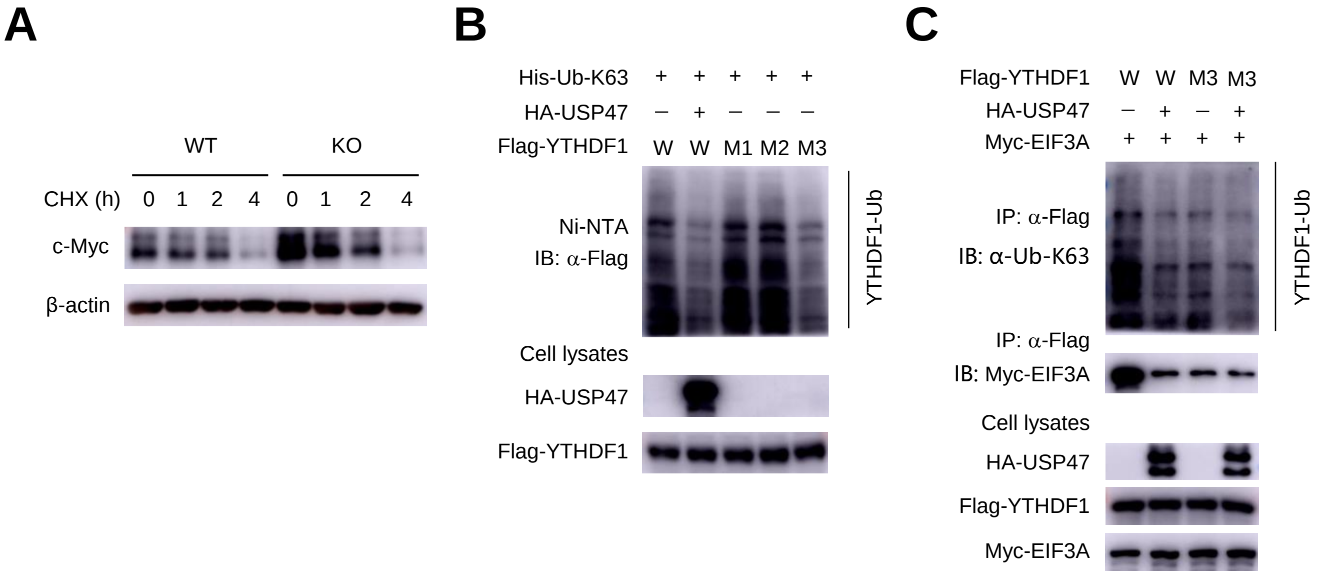

Supplementary Figure 4. USP47 prevents YTHDF1 K63-linked ubiquitination of lysine 500 (K500). (A) Immunoblot analysis of the indicated proteins in Treg cells stimulated with anti-CD3 and anti-CD28 antibodies for 2 hours following incubation with CHX (50 μg/mL) for the indicated durations. **(B)** Flag-tagged mouse YTHDF1 or its mutant variants were transfected into HEK293T cells for YTHDF1 K63-linked ubiquitination assays. W, WT-YTHDF1; M1, K370R; M2, K372R; M3, K500R. **(C)** Indicated plasmids were transfected into HEK293T cells for YTHDF1 K63-linked ubiquitination and YTHDF1-EIF3A interaction assays. W, WT-YTHDF1; M3, K500R-YTHDF1. The data shown are representative of three independent experiments.

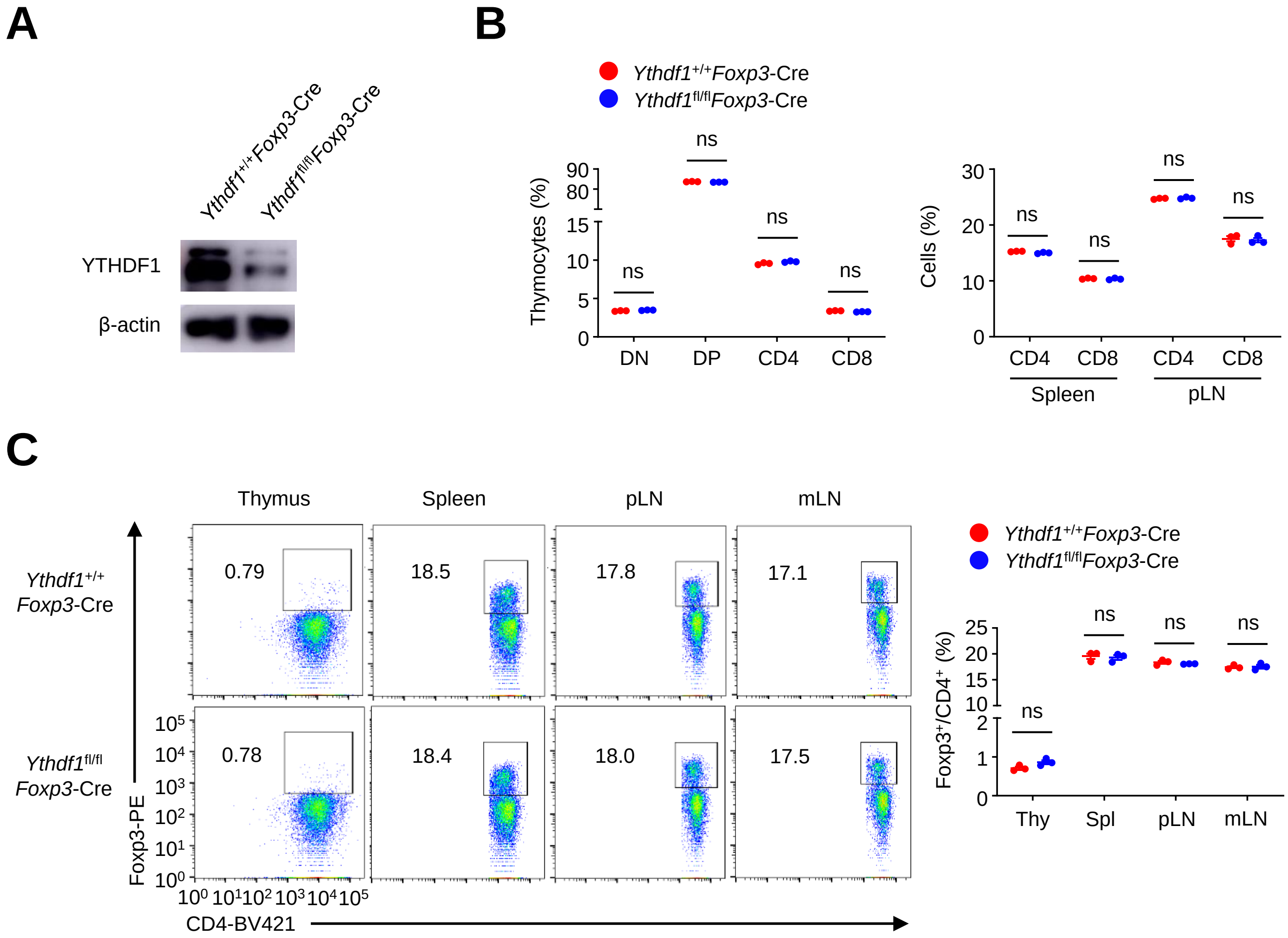

Supplementary Figure 5. T cell development and homeostasis in *Ythdf1*$^{fl/fl}$*Foxp3*-Cre mice. (A) IB analysis of YTHDF1 using isolated $CD4^+YFP^+$ cells from *Ythdf1*$^{+/+}$*Foxp3*-Cre (WT) and *Ythdf1*$^{fl/fl}$*Foxp3*-Cre (KO) mice. **(B)** Flow cytometric analysis of the percentage of T cells in the thymus, spleen and peripheral lymph node (pLN) of 6-week-old *Ythdf1*$^{+/+}$*Foxp3*-Cre and *Ythdf1*$^{fl/fl}$*Foxp3*-Cre mice (n=3). **(C)** Flow cytometric analysis of the percentage of $CD4^+Foxp3^+$ T cells in the thymus (Thy), spleen (Spl), pLN and mLN of 6-week-old *Ythdf1*$^{+/+}$*Foxp3*-Cre and *Ythdf1*$^{fl/fl}$*Foxp3*-Cre mice (n=3). Data are representative of more than three independent experiments and are presented as means ±SEM. ns, not statistically significant. Two-tailed Student's *t* test.
